# New Insights into the Opioid Analgesic Profile of *cis*-(−)-*N*-Normetazocine-derived Ligands

**DOI:** 10.3390/molecules28124827

**Published:** 2023-06-17

**Authors:** Giuliana Costanzo, Rita Turnaturi, Carmela Parenti, Salvatore Spoto, Silvia Piana, Maria Dichiara, Chiara Zagni, Anna Rita Galambos, Nariman Essmat, Agostino Marrazzo, Emanuele Amata, Mahmoud Al-Khrasani, Lorella Pasquinucci

**Affiliations:** 1 Department of Biomedical and Biotechnological Sciences, University of Catania, Via Santa Sofia 97, 95123 Catania, Italy; giuliana.costanzo93@gmail.com; 2 Department of Drug and Health Sciences, Medicinal Chemistry Section, University of Catania, Viale A. Doria 6, 95125 Catania, Italy; rita.turnaturi@unict.it (R.T.); silviapiana@outlook.com (S.P.); mariadic.md@gmail.com (M.D.); chiara.zagni@unict.it (C.Z.); marrazzo@unict.it (A.M.); eamata@unict.it (E.A.); 3 Department of Drug and Health Sciences, Section of Pharmacology and Toxicology, University of Catania, Viale A. Doria 6, 95125 Catania, Italy; cparenti@unict.it (C.P.); salvospoto12@icloud.com (S.S.); 4 Department of Pharmacology and Pharmacotherapy, Faculty of Medicine, Semmelweis University, Nagyvárad tér 4, H-1089 Budapest, Hungary; galambos.anna@phd.semmelweis.hu (A.R.G.); nariman.gomaa@phd.semmelweis.hu (N.E.)

**Keywords:** μ opioid receptor, competition binding assay, mouse vas deferens assay, tail-flick test, CFA test

## Abstract

In this work, we report on the in vitro and in vivo pharmacological properties of LP1 analogs to complete the series of structural modifications aimed to generate compounds with improved analgesia. To do that, the phenyl ring in the *N*-substituent of our lead compound LP1 was replaced by an electron-rich or electron-deficient ring and linked through a propanamide or butyramide spacer at the basic nitrogen of the (−)-*cis*-*N*-normetazocine skeleton. In radioligand binding assays, compounds **3** and **7** were found to display nanomolar binding affinity for the μ opioid receptor (MOR) (K_i_ = 5.96 ± 0.08 nM and 1.49 ± 0.24 nM, respectively). In the mouse vas deferens (MVD) assay, compound **3** showed an antagonist effect against DAMGO ([D-Ala^2^, N-MePhe^4^, Gly-ol]-enkephalin), a highly selective MOR prototype agonist, whereas compound **7** produced naloxone reversible effect at MOR. Moreover, compound **7**, as potent as LP1 and DAMGO at MOR, was able to reduce thermal and inflammatory pain assessed by the mouse tail-flick test and rat paw pressure thresholds (PPTs) measured by a Randall–Selitto test.

## 1. Introduction

**Figure 1:**
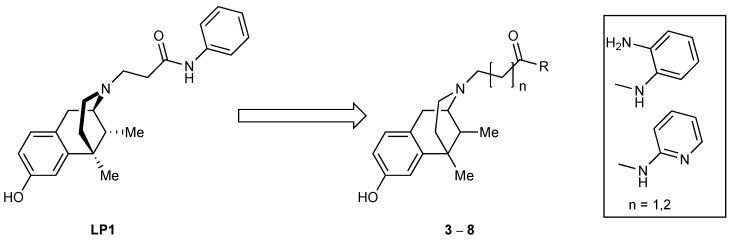
Chemical structures of LP1 and newly synthesized compounds.

The modification of the *N*-substituent in classical opioid-type structures, such as 4,5-epoxymorphinans, morphinans, 6,7-benzomorphans, and 5-phenylmorphans, affects binding, selectivity, potency, and efficacy at opioid receptors. A variety of compounds have been synthesized based on the skeleton of (−)-*cis*-*N*-normetazocine, and most of the modifications happened in the *N*-substituent and/or phenolic hydroxyl group [1]. As part of ongoing efforts to develop safer analgesics, Pasquinucci et al. synthesized several series of (−)-*cis*-*N*-normetazocine derivatives by modifying the *N*-substituent [2]. In this context, LP1, a μ opioid receptor (MOR) agonist/δ opioid receptor (DOR) antagonist ligand (Figure 1) [3], was found to be able to alleviate nociceptive pain and behavioral signs of persistent pain with low tolerance-inducing capability [4,5]. The LP1 ligand is featured by a *cis*-(−)-*N*-normetazocine skeleton with an *N*-phenylpropanamide substituent that was demonstrated to have a crucial role. Building upon the related structure–activity relationship (SAR) information, other chemical series were synthesized in an effort to optimize the physicochemical and biological properties of the lead LP1. Since minor structural modifications often result in significant changes in the pharmacological profile of opioid ligands, we expanded our SARs evaluating the importance of the nature [3], size [6], hydrophobic [7], electronic [8], and steric [9] properties of the *N*-substituent in the achievement of the proper pharmacological fingerprint. These efforts provided several pharmacologically interesting compounds [10,11,12].

In this paper, we describe a representative strategy to obtain LP1 analogs by targeting the *N*-substituent and to expand the SARs of this series of ligands **(**Figure 1). The focus was on the key aromatic ring that was replaced by an electron-rich (compounds **3** and **5**) or an electron-deficient (compounds **7**) analog ring. Moreover, a methylene-extended alkyl linker was further inserted in compound **5**. The *N*-substituent modifications described were maintained in compounds **4**, **6**, and **8** with a *cis*-(+)-*N*-normetazocine skeleton to parallel these latter with levo compounds (**3**, **5**, and **7**, respectively) and to evaluate the stereochemistry impact on the isomer–target interaction.

Here, we report on the in vitro and in vivo evaluations of new synthesized LP1 analogs. In order to investigate the affinity and selectivity of new compounds, competition binding assays on opioid and sigma receptors were performed. Selected compounds with significant inhibitory effects were further screened for their efficacy profile by ex vivo mouse vas deferens (MVD) assay. Finally, the antinociceptive effect of the most promising compound was determined by tail-flick and complete Freund’s adjuvant (CFA)–evoked hyperalgesia assays.

## 2. Results 

### 2.1. Chemistry

**Scheme 1:**
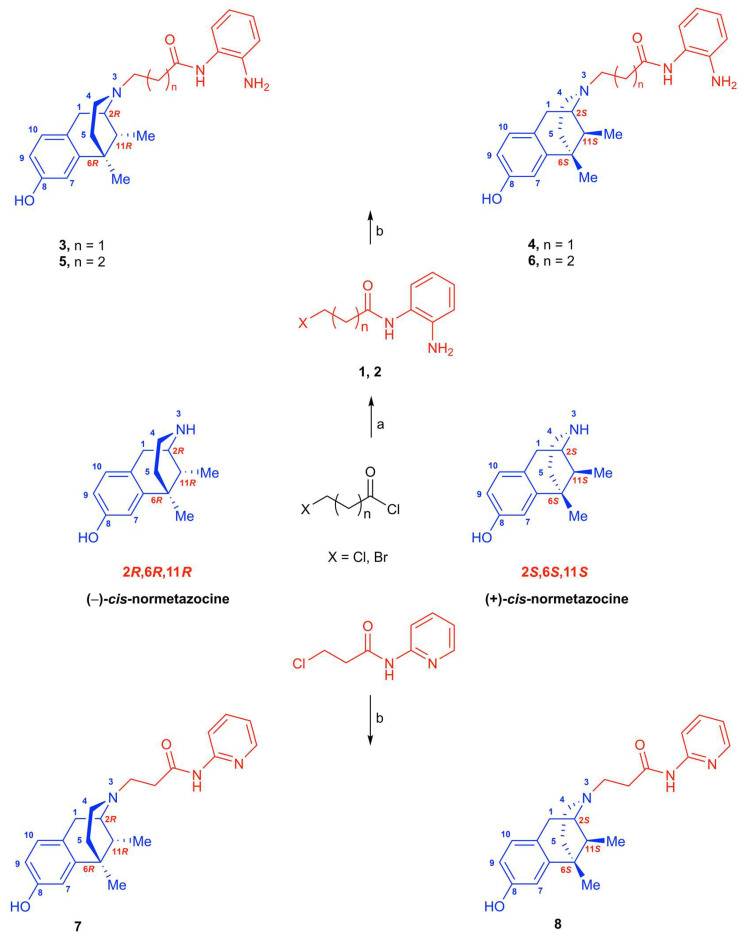
Synthesis of novel LP1 analogs. Reagents and conditions: (**a**) *o*-phenylenediamine, anhydrous CH_2_Cl_2_, 0 °C, nitrogen atmosphere, RT, 3 h; (**b**) **1**, **2**, or 3-chloro-*N*-(pyridin-2-yl)propanamide, NaHCO_3_, KI, DMF, 55 °C, 72 h.

(−)-*Cis*-*N*-normetazocine and (+)-*cis*-*N*-normetazocine were obtained through the resolution of (±)-*cis*-*N*-normetazocine as previously reported [13]. Amides **1** and **2** were prepared by amine acylation in anhydrous CH_2_Cl_2_ at 0 °C with 3-bromopropionyl chloride and 4-chlorobutyryl chloride, respectively. Target compounds **3**–**8** were synthesized by alkylation of *cis*-(−)-*N*-normetazocine or *cis*-(+)-*N*-normetazocine with the respective amides **1, 2**, or 3-chloro-*N*-pyridin-2-ylpropanamide in DMF at 55 °C in the presence of NaHCO_3_ and KI (Scheme 1). All newly synthesized compounds were characterized by ^1^H NMR, ^13^C NMR, MALDI-TOF spectroscopy and elemental analysis (Supplementary Materials).

### 2.2. Radioligand Binding Assays

Using a radioligand binding assay, compounds **3**–**6** and **7**–**8** were screened vs. MOR, DOR, κ opioid receptor (KOR), sigma-1 and -2 receptors (σ1R and σ2R), according to the procedures reported previously with minor revisions [14,15]. LP1, DAMGO, naltrindole, (−)-U50,488, haloperidol, (+)-pentazocine, DTG, and BD-1063 were tested under the same conditions for comparison (Table 1). 

Compounds **3** and **7**, with an *o*-phenylenediamine or *o*-aminopyridine moiety in the *N*-substituent of *cis*-(−)-*N*-normetazocine scaffold, retained high binding affinity at MOR within the nanomolar range, confirming a key π-interaction with the binding site of MOR. The importance of this interaction was underlined in compound **5**, in which an extended alkyl linker resulted in a poorly MOR binding interaction. With respect to the DOR affinity of LP1, a worsened profile was assayed in compounds **3** and **7**, with a parallel decrement of affinity in compound **5**. Similarly, KOR affinity was lost in compound **5** but retained in compounds **3** and **7**, showing K_i_ values in the same nanomolar range of LP1. Concerning the MOR selectivity, it thus seemed that electron-rich and electron-deficient rings in compounds **3** and **7** gave a lower MOR selectivity over the KOR and in compound **7** improved MOR selectivity over DOR, relative to LP1. On the other hand, compounds **4**, **6,** and **8**, with a (+)-*N*-normetazocine skeleton, have provided to displace the opioid receptors’ radioligands only at high concentrations (0.5–1 micromolar range), resulting in a low affinity profile for all opioid receptor subtypes. Moreover, none of the synthesized compounds, with either a (−)-*cis*-*N*-normetazocine or a (+)-*cis*-*N*-normetazocine nucleus, showed affinities for σ1R and σ2R. 

### 2.3. Evaluation of Opioid Activity in Mouse Vas Deferens

To further explore the functional significance of compounds **3** and **7**, which showed promising MOR binding profiles, MVD assay was performed. 

**Figure 2:**
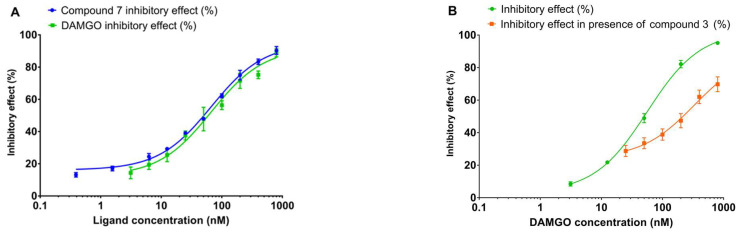
(**A**): The inhibitory concentration effect curve of compound **7** on electrically evoked contractions of MVD, compared with DAMGO. Results are plotted as mean ± SEM. The EC_50_ values are presented in Table 1. (**B**): The rightward shift of the DAMGO concentration–response curve in the presence of 400 nM compound **3**. Results are plotted as mean ± SEM. The IC_50_ values are presented in Table 2.

**Figure 3:**
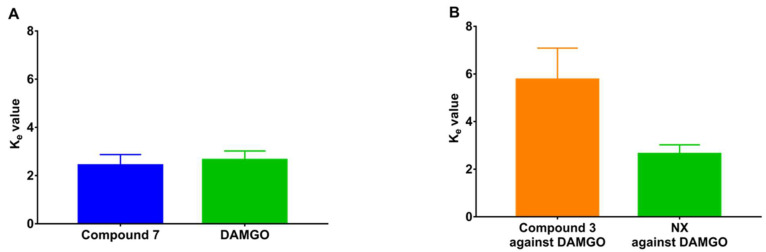
Unpaired T-test between K_e_ values (single-dose method) of compound **7** and DAMGO in the presence of naloxone (**A**) and compound **3** and naloxone against DAMGO (**B**).

As depicted in Figure 2A, compound **7** showed a concentration-dependent inhibitory effect on the electrically evoked MVD smooth muscle contractions. The calculated EC_50_ was comparable to DAMGO (Table 2), indicating that compound **7** has a strong potency similar to DAMGO, a highly selective and high-efficacy prototype MOR agonist. In addition, the T-test between the calculated K_e_ value of naloxone against DAMGO and compound **7** (Figure 3A, Table 2) reveals that the produced effect is mediated by MOR. Thus, these data indicate the higher functional selectivity of compound **7** for MOR, yet it shows low selectivity at KOR and negligible selectivity at DOR (Table 2). The opioid receptor preference was assessed in the MVD assay in the presence of naloxone (nonselective opioid antagonist), naltrindole (selective DOR antagonist), or *nor*-BNI (selective KOR antagonist). On the other hand, compound **3** emerged at MVD as an antagonist, indicated by its ability to shift to the right (DR = 5) the concentration–response curve of DAMGO and produce an affinity of 5.76 ± 1.32 nM against DAMGO (Figure 2 and Figure 3B).

### 2.4. Thermal Acute Pain Model (Tail-Flick Test) 

**Figure 4:**
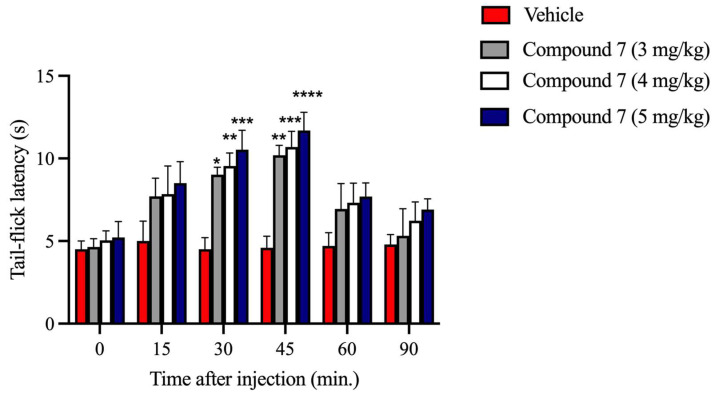
Antinociceptive effect of compound **7** at different time points of the tail-flick test. Intraperitoneal (i.p.) administration of compound **7** (3, 4, and 5 mg/kg) showed a significant increase in latency after 30 and 45 min of injection, compared with the vehicle group, in the mouse tail-flick test. Data are expressed as MEAN ± SEM (*n* = 6–8 per group). **** *p* < 0.0001, *** *p* < 0.0005, ** *p* < 0.005, * *p* < 0.05 vs. Vehicle, two-way ANOVA, followed by Sidak’s multiple comparison test.

Based on competition binding and MVD functional assay data, the most interesting compound **7** was further tested for its antinociceptive profile [10]. The effect of compound **7**, intraperitoneally (i.p.) administrated, was evaluated at three doses by the tail-flick test, as shown in Figure 4. The pretreatment of mice with compound **7** (at doses of 3, 4, and 5 mg/kg) increased the mean tail-flick latency compared with the group of animals treated with vehicle. A significant antinociceptive effect was obtained starting at 30 min and reached the highest value (**** *p* < 0.0001 vs. vehicle group) at 45 min after i.p. injection.

### 2.5. The Antinociceptive Effects of Compound **7** after s.c. Administration in Inflamed and Noninflamed Rat Paws

**Figure 5:**
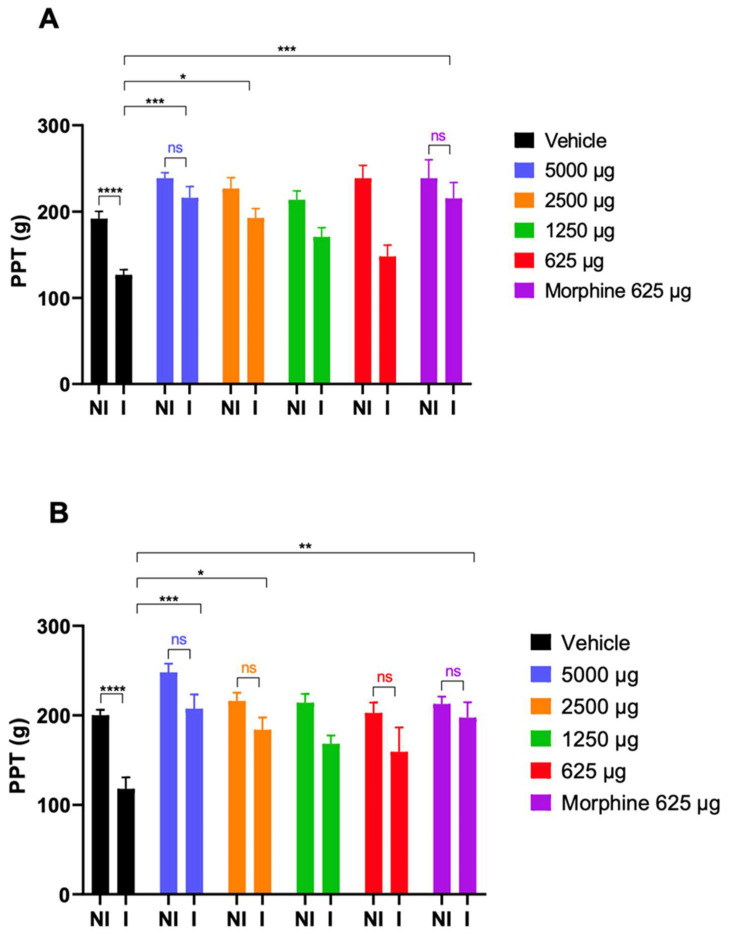
The antinociceptive effects of s.c. administered compound **7** (µg/kg, *n* = 5–10/group) as measured by PPT via the Randall–Selitto assay in rats. Drugs were delivered at a volume of 5 mL/kg body weight. Each value represents the mean ± SEM. Data were obtained 30 (panel (**A**)) and 60 min (panel (**B**)) after injection of compound **7**, morphine, or vehicle (DMSO + saline). Statistical analysis was performed by two-way ANOVA, followed by Sidak’s post hoc test. A *p* < 0.05 is considered significant. Asterisks mark the significant differences compared with the vehicle group. ****: *p* < 0.0001; ***: *p* < 0.0003; ** *p* < 0.002; *: *p* < 0.02; ns: nonsignificant. I: inflamed paw; NI: noninflamed paw.

The intraplantar (i.pl.) injection of CFA resulted in a significant decrease in the mean of the paw pressure threshold (PPT) of the right hind paws (211 ± 34 vs. 150 ± 40 g, *n* = 55, *p* < 0.0001) 4 days after injection. In contrast, no change was observed in the left paw PPT (210 ± 34 vs. 217 ± 35 g, *n* = 55, *p* = 0.1835). Next, animals were further divided into 6 groups (5–10 per group). Within these groups, the right paw PPT was significant compared with the left paw before drug administration. As shown in to Figure 5A,B, compound **7** was tested in the dose range of 625–5000 µg/kg for its ability to restore hyperalgesia of inflamed paws. Compound **7** proved to be effective at 30 and 60 min in the higher tested doses (5000 and 2500 µg/kg), whereas the lower doses showed a tendency of antinociceptive effect. Furthermore, in certain doses, compound **7** could also significantly increase the noninflamed PPT of animals compared with those treated with the vehicle. Morphine was used as a positive control.

## 3. Discussion 

Opioid analgesics relieve pain by activating opioid receptors (MOR, DOR, and KOR), and among them, MOR mediates the effects of most clinically relevant opioid analgesics. Several efforts have been made to promote the development of potent but safer opioid analgesics by the continuous exploring of the underlying mechanisms of MOR signaling. Among them, developing bifunctional or multifunctional drugs that simultaneously modulate MOR and one or more other targets has attracted attention [2]. 

Here, we report on the chemistry and pharmacology of novel *N*-normetazocine (benzomorphan)-based compounds, namely, (−)-*cis*-*N*-normetazocine- or (+)*-cis*-*N*-normetazocine-based analogs. The (−)-*cis*-*N*-normetazocine-based analog like LP1 has *N*-phenylpropanamido substituent linked to the (−)-*cis*-*N*-normetazocine scaffold [3]. The structure of the novel compounds differs from LP1 in the nature of the aromatic ring in *N*-substituent, phenyl vs. *o*-aminopyridine moiety or *o*-phenylenediamine moiety, as well as in the length of the chain connecting *o*-phenylenediamine moiety to the (−)-*cis*-*N*-normetazocine or (+)-*cis*-*N*-normetazocine (compounds **3**–**8**). However, the majority of the novel compounds reported here have a propanamido connecting chain (compounds **3**, **4**, **7**, and **8**). 

In the first part of the work, the synthesis and receptor binding profiles of all of the above-mentioned novel compounds (**3**–**8**) were thoroughly described. In the receptor binding assay, the results indicated that compounds bearing *o*-aminopyridine moiety or *o*-phenylenediamine moiety, compounds **7** and **3**, respectively, displayed high affinity in the 10 nM range for MOR. Additionally, compound **7** showed the highest affinity for both MOR and KOR. In comparison with LP1, compound **7** showed higher affinity for KOR. On the other hand, LP1 proved to have a higher affinity for both MOR and DOR in the previous studies carried out by our group [3]. As stated above, LP1 is a (−)-*cis*-*N*-normetazocine-based compound bearing a phenylpropanamide substituent at the basic nitrogen [3], whereas compounds **7** and **3** have *o*-aminopyridine moiety or *o*-phenylenediamine in the *N*-substituent. Several previous studies have been intended to address the impacts of chemical modification on the *N*-substituent of the normetazocine scaffold on opioid affinity, potency, and receptor selectivity for the three opioid receptor subtypes. In this regard, in a previous work where the primary amide substituent was changed from phenyl (LP1) to cyclohexyl ring, dramatic change in MOR affinity was observed, namely, a 60-fold increase in K_i_ value (K_i_ = 56 nM) [3]. Furthermore, the replacement of phenyl with bulkier rings, such as 1- or 2-naphthyl, quinolines, isoquinoline, indoline, diphenylamine, tetrahydroquinoline, caused a shift in the MOR efficacy profile from agonist to antagonist [6,8]. In particular, the 1-naphthyl propanamide substituent at the basic nitrogen resulted in a selective and potent MOR antagonist with a K_i_ of 38 nM and an antagonist affinity (pA_2_) of 8.6 nM [6].

The *para* electron-donating or electron-withdrawing groups’ insertion in the LP1 phenyl ring negatively altered the opioid binding profile in all obtained compounds, especially with respect to LP1 affinity for MOR, highlighting a negative steric hindrance. The LP1 analog with a *p*-fluorophenyl in the *N*-substituent only shared the functional profile of LP1, although it was less potent [8]. Analogously, the phenyl ring alkylation (in various ways and positions) revealed that *para*-methylation was unfavorable, while *ortho-* and *meta*-methylation was well tolerated [9]. Indeed, these later compounds retained the opioid receptor affinity profile of LP1.

Next, in compounds with *N*-methyl- or *N*-ethyl-*N*-phenyl-propanamide chains, the introduction of a secondary amide decreased their MOR affinity as a function of the steric bulk of the alkyl amide substituents (K_i_ = 65 and 136 nM, respectively) [3]. Additionally, the introduction of a benzyl pendant at the amidic nitrogen, with and without simultaneous phenyl ring methylation, induced detrimental changes in the opioid binding affinity of all relative compounds, confirming an impediment in receptor interaction [9].

On the other hand, only the amide functionality substitution with more flexible ethylamino and propylamino *N*-substituents allowed all these LP1 analogs to reposition themselves relative to the (−)-*cis*-*N*-normetazocine nucleus, thus producing a different pharmacological profile at MOR, DOR, and KOR. Their second positive charge at the *N*-substituent retained MOR affinity, but increased KOR affinity and dramatically reduced DOR affinity. For instance, the 2-methyl-phenyl-ethylamino LP1 analog displayed a full MOR agonist both in in vitro and in vivo assays [7].

Shortening the length of the chain between the phenyl ring and the (−)-*cis*-*N*-normetazocine nucleus resulted in a dramatic loss of affinity for opioid receptors, as shown for the resulting acetamido analogs. On the other hand, the attempts to increase this distance failed to improve the MOR affinity in the *N*-benzyl-propanamide analog of LP1 (Ki = 105 nM) [3]. 

Likewise, the shorter and flexible *N*-substituent in the (*R*/*S*)-2-methoxy-2-phenyl-ethyl derivative named LP2 maintained the opioid receptors’ affinity of LP1 compound but shifted its efficacy profile at DOR from antagonist to agonist, in comparison with LP1 [10]. Interestingly, the (2*S*)-2-methoxy-2-phenyl-ethyl analog, featured by the same pharmacodynamic profile, was endowed with functional selectivity, thus resulting in a biased MOR/DOR agonist [11]. 

Taken together, the binding data of the novel compounds (**3**–**8**) reveal that (a) the aromatic ring is required for nanomolar affinity to MOR, although with a slight preference for the electron-deficient ring analog, the o-substituted pyridine; (b) the distance with (−)-*cis*-*N*-normetazocine nitrogen is important probably due to a misplacement of the aromatic ring, resulting in the loss of possible interaction; (c) compounds **3** and **7** showed reduced KOR/MOR selectivity with respect to LP1 due to a better KOR affinity profile; and (d) compounds with a (+)-*cis*-*N*-normetazocine skeleton are not able to bind σ1R and σ2R, probably due to unfavorable interactions of their N-substituents with the respective binding pockets. In addition, competition binding data corroborated the importance of an aromatic ring and its optimal distance by a (−)-*cis*-*N*-normetazocine nucleus for ligand–MOR interaction.

Although both electron-rich and electron-deficient rings bind MOR with similar K_i_ and profile of selectivity with respect to DOR and KOR, their different electronic properties differently stabilize MOR conformation with a consequently different signaling pathway. The (−)-*cis*-*N*-normetazocine nucleus, originated from morphine structure simplification, provides a rigid backbone represented by the aromatic ring, the saturated or morphan segment, and the nitrogen substituent identical to that of (−)-morphine. Moreover, as different properties of its *N*-substituent affect the achievement of a specific affinity vs. opioid receptor subtypes and determine specific functional profiles [1], various SARs have been conducted. First, May et al., in a series of *N*-alkyl-substituted (−)-*cis*-*N*-normetazocines, demonstrated the correlation between their affinity values and chain elongation. Moreover, a switch of activity from agonist (*N*-methyl) to agonist/antagonist (*N*-ethyl, -propyl and -butyl) and back to agonist (*N*-pentyl, -hexyl, -heptyl, and -octyl) until inactivity (*N*-nonyl and -decyl) was detected [14]. In a next series of *N*-alkenyl-, *N*-alkynyl-, and *N*-cyanoalkyl-substituted analogs, an optimal chain length was assayed in affinity and potency profiles [15]. To study the influence of *N*-substituent lipophilicity/hydrophilicity in opioid receptor interaction, Metcalf et al. [16] synthesized different compounds bearing at *N*-substituent ester or carboxylic acid groups. Both ester and carboxylic acid derivatives showed K_i_ values for all opioid receptors in the micromolar range. Moreover, the spacer length of the *N*-substituent changed in in vitro activity. In the *N*-acid derivatives, the elongation of spacer length reduced activity, while in the N-ester series, it was observed as a switch of activity from antagonist to agonist.

Compound **7** with *o*-aminopyridine moiety in the *N*-substituent of the (−)-*cis*-*N*-normetazocine scaffold showed a promising opioid property profile, which encouraged us to further investigate it in MVD and acute thermal and subchronic inflammatory pain assays. Compared with (D-Ala^2^, MePhe^4^, Gly^5^-ol)-enkephalin (DAMGO), a highly selective MOR agonist peptide [17], compound **7** showed a similar affinity for the MOR in a receptor binding assay. In MVD, compound **7** and DAMGO displayed comparable potency. It is noteworthy that naloxone’s K_e_ values obtained by the same assay were also comparable, indicating the MOR mediating action. Nonetheless, compound **7** showed a weak KOR- and no measurable DOR-mediated effect. Indeed, MVD hosts all opioid receptor subtypes with the highest reserve for DOR and the lowest for KOR [18]. Therefore, compound **7** activates both MOR and KOR. Opioid ligands that activate MOR and KOR are used to manage mild to severe acute and chronic pains. Based on the MVD and competition binding assays’ results, the second task in the present study was to investigate the impact of compound **7** on pain evoked by thermal stimulus or complete Freund’s adjuvant (CFA) that models acute thermal or subchronic inflammatory pain, respectively [19,20]. The tail-flick test is one of the well-known pain assays for measuring the antinociceptive effect of opioids. In this pain model, compound **7** produced a dose-dependent antinociceptive effect that peaked at 30–45 min following i.p. injection. Likewise, it significantly attenuated the CFA-evoked hyperalgesia of inflamed paws at 30 and 60 min after s.c. administration. Previous works have identified that LP1, which shares a similar structure with compound **7**, is able to address both nociception and behavioral signs of persistent pain conditions with low tolerance-inducing capability [4,5]. However, when we consider the receptor profiles of the two compounds, LP1 showed MOR and DOR binding preference, whereas compound **7** showed MOR and KOR binding preference. It means that both activate MOR, which is the main target of the majority of current opioid analgesics used in clinical practice and also causes opioid use disorder, which creates complex social problems that need solutions. One of the strategies to decrease opioid addiction is to decrease addiction liability by developing opioids with lower abuse potential. In this regard, previous works have shown that drugs activating KOR displayed lower abuse liability yet exerted an inverse effect on morphine-induced side effects [21,22]. This opioid property is likely hosted by compound **7,** and to elucidate it, future studies are needed. 

## 4. Materials and Methods

### 4.1. General Remarks

Reagent-grade chemicals were purchased as previously described [23]. Melting points were determined in open capillary tubes with a Büchi 530 apparatus and are uncorrected. Optical rotations were determined in EtOH solution with a PerkinElmer 241 polarimeter. ^1^H and ^13^C NMR spectra were recorded at 200 and 500 MHz on Varian Inova spectrometers in CDCl_3_ or DMSO-d_6_. Chemical shifts δ are expressed in parts per million (ppm) with reference to tetramethylsilane as an internal standard. MALDI mass spectra were acquired in reflector mode by a 4800 MALDI TOF/TOF™ Analyzer (Applied Biosystems, Framingham, MA, USA). The excitation source is an Nd:YAG laser (wavelength of 355 nm) <500 ps pulse and 200 Hz repetition rate and working in positive-ion mode. The mass resolution of MALDI spectra was about 10,000 (full width at half maximum, FWHM), and the mass accuracy was 1–10 ppm for masses in the range *m*/*z* 200–1000 Da. *Trans*-2-[3-(4-tert-Butylphenyl)-2-methyl-2-propenyldene] malononitrile (0.1 mmol in THF) was used as a matrix and mixed with appropriate volumes of samples dissolved in methanol (10 mg/mL concentration) at 1:1, 1:2, and 2:1 ratios (sample/matrix *v*/*v*). An amount of 1 µL of each sample/matrix mixture was spotted onto the MALDI sample holder and dried at 25 °C to allow matrix crystallization. The structural identification of MALDI peaks was mainly made based on empirical formulas. Elemental analyses (C, H, N) were performed on a Carlo Erba 1106 analyzer, and the results were within ±0.4% of the theoretical values. 

### 4.2. Preparation of Amides ***1*** and ***2***

*N-(2-aminophenyl)-3-bromopropanamide* (**1**). *o*-Phenylenediamine (9.24 mmol, 3 eq) was dissolved in CH_2_Cl_2_ (10 mL), the solution was cooled at 0 °C under nitrogen atmosphere, and 3-bromopropionyl chloride (3.08 mmol, 1 eq) was added dropwise. After 3 h at RT, the reaction mixture was quenched with H_2_O and extracted with CH_2_Cl_2_. The organic phase was washed with brine, dried over anhydrous Na_2_SO_4_, and concentrated in vacuo. The crude product was purified by flash chromatography on silica gel using CH_2_Cl_2_/EtOAc/EtOH (50:48:2 *v*/*v*), as solvents to obtain a white solid. Yield: 49%; Mp: 195–196 °C; TLC CH_2_Cl_2_/EtOAc/EtOH (50:48:2 *v*/*v*) R_f_ = 0.46; ^1^H NMR (200 MHz, CDCl_3_): δ = 7.25–7.20 (m, 2H); 7.14–7.06 (m, 1H); 6.84–6.77 (m, 1 H); 3.75 (t, 2H, *J* = 6.2 Hz); 2.99 (t, 2H, *J* = 6.2 Hz).

*N-(2-aminophenyl)-4-chlorobutanamide* (**2**). *o*-Phenylenediamine (9.24 mmol, 3 eq) was dissolved in CH_2_Cl_2_ (10 mL), the solution was cooled at 0 °C under nitrogen atmosphere, and 4-chlorobutyryl chloride (3.08 mmol, 1 eq) was added dropwise. After 3 h at RT, the reaction mixture was quenched with H_2_O and extracted with CH_2_Cl_2_. The organic phase was washed with brine, dried over anhydrous Na_2_SO_4_, and concentrated in vacuo. The crude product was purified by flash chromatography on silica gel using CH_2_Cl_2_/EtOAc/EtOH (50:48:2 *v*/*v*), as solvents to obtain a white solid. Yield: 51%; Mp: 192–193 °C; TLC CH_2_Cl_2_/EtOAc/EtOH (50:48:2 *v*/*v*) R_f_ = 0.50; ^1^H NMR (200 MHz, CDCl_3_): δ = 7.18–7.17 (m, 1H); 7.06–7.03 (m, 1H); 6.79–6.77 (m, 2H), 3.66 (t, 2H, *J* = 6.0 Hz); 2.56 (t, 2H, *J* = 6.0 Hz); 2.18 (q, 2H, *J* = 6.0, 6.0 Hz).

### 4.3. Preparation of Target Compounds ***3***–***8***

*cis-(−)-N*-normetazocine or *cis-(+)-N*-normetazocine (1 eq) was dissolved in DMF (5 ml), and the appropriate amide **1**–**2** or 3-chloro-*N*-pyridin-2-ylpropanamide (1.5 eq), NaHCO_3_ (1.5 eq), and a catalytic amount of KI were added. The reaction mixture was stirred for 72 h at 55 °C. After cooling, the reaction mixture was filtered and concentrated under vacuum to remove DMF. The resulting residue was purified by flash chromatography on a silica gel column using CH_2_Cl_2_/EtOH (95:5 *v*/*v*) as an eluent to obtain a dark solid.

*N*-(2-aminophenyl)-3-((2*R*,6*R*,11*R*)-8-hydroxy-6,11-dimethyl-1,4,5,6-tetrahydro-2,6-methanobenzo[d]azocin-3(2H)-yl)propanamide (**3**). Yield = 36%; Mp: 160–163 °C; ${\left[\alpha \right]}_{D}^{25}$ = –60.5° (c 1.004, EtOH); TLC CH_2_Cl_2_/EtOH (95:5 *v*/*v*) R_f_ = 0.22; ^1^H NMR (CDCl_3_): δ = 10.88 (s, 1H, exchangeable in D_2_O); 7.10–7.03 (m, 2H); 6.84–6.79 (m, 3H); 6.71–6.70 (m, 1H) 6.64–6.63 (m, 1H); 3.07–2.78 (m, 6H); 2.72–2.57 (m, 4H); 2.23–2.16 (m, 1H); 1.92–1.78 (m, 1H); 1.31 (t, 3H); 0.85 (d, 3H); ^13^C (CDCl_3_): δ = 171.3; 154.6; 142.1; 140.5; 128.2; 126.9; 126.5; 124.5; 124.3; 118.9; 117.9; 113.0; 112.2; 57.8; 50.4; 44.8; 41.9; 36.2; 32.5; 29.7; 25.8; 24.4; 13.9. Mass: *m*/*z* calc C_23_H_29_N_3_O_2_ 379.2 [M + H]^+^; found 380.3. Anal (C_23_H_29_N_3_O_2_) C, H, N (Table S1).

*N*-(2-aminophenyl)-3-((2*S*,6*S*,11*S*)-8-hydroxy-6,11-dimethyl-1,4,5,6-tetrahydro-2,6-methanobenzo[d]azocin-3(2H)-yl)propanamide (**4**). Yield = 35%; Mp = 160–163 °C; ${\left[\alpha \right]}_{D}^{25}$ = +61.0° (c 1.002, EtOH); TLC CH_2_Cl_2_/EtOH (95:5 *v*/*v*) R_f_ = 0.22; ^1^H NMR (CDCl_3_): δ = 10.81 (s, 1H, exchangeable in D_2_O); 7.03–6.96 (m, 2H); 6.86–6.74 (m, 3H); 6.64–6.63 (m, 1H); 6.57–6.53 (m, 1H); 3.00–2.71 (m, 6H); 2.64–2.50 (m, 4H); 2.15–2.09 (m, 1H); 1.86–1.71 (m, 1H); 1.24 (t, 3H); 0.78 (d, 3H); ^13^C (CDCl_3_): δ = 171.5; 154.7; 142.3; 140.6; 128.4; 127.0; 126.7; 124.7; 124.5; 119.1; 118.1; 113.2; 112.3; 58.0; 51.0; 45.0; 42.0; 36.4; 32.7; 29.9; 26.0; 24.5; 14.1. Mass: *m*/*z* calc C_23_H_29_N_3_O_2_ 379.2 [M + H]^+^; found 380.2. Anal (C_23_H_29_N_3_O_2_) C, H, N (Table S1).

*N*-(2-aminophenyl)-4-((2*R*,6*R*,11*R*)-8-hydroxy-6,11-dimethyl-1,4,5,6tetrahydro-2,6-methanobenzo[d]azocin-3(2H)-yl)butanamide (**5**). Yield = 38%; Mp = 155–158 °C; ${\left[\alpha \right]}_{D}^{25}$ = −58.7° (c 1.001, EtOH); TLC CH_2_Cl_2_/EtOH (95:5 *v*/*v*) R_f_ = 0.32); ^1^H NMR (DMSO_d6_): δ = 11.18 (s, 1H, exchangeable in D_2_O); 6.50–6.41 (m, 4H); 6.13–6.11 (m, 3H); 3.64 (br, 2H); 2.49–2.36 (m, 5H); 1.88–1.80 (m, 3H); 1.52–1.51 (m, 9H); 0.95–0.88 (m, 3H); ^13^C (DMSO_d6_): δ = 169.9; 155.0; 144.6; 140.5; 121.2; 121.1; 121.0; 120.7; 120.4; 118.0;117.6; 112.6; 110.4; 60.1; 47.1; 40.6; 40.2; 39.8; 39.4; 30.8; 29.0; 25.3; 22.12; 10.8. Mass: *m*/*z* calc C_24_H_31_N_3_O_2_ 393.2 [M + H]^+^; found 394.4. Anal (C_24_H_31_N_3_O_2_) C, H, N (Table S1).

*N*-(2-aminophenyl)-4-((2*S*,6*S*,11*S*)-8-hydroxy-6,11-dimethyl-1,4,5,6tetrahydro-2,6-methanobenzo[d]azocin-3(2H)-yl)butanamide (**6**). Yield = 60%; Mp = 157–160 °C; ${\left[\alpha \right]}_{D}^{25}$ = +57.7° (c 1.00, EtOH); TLC CH_2_Cl_2_/EtOH (95:5 *v*/*v*) R_f_ = 0.30); ^1^H NMR (DMSO_d6_): δ = 11.18 (s, 1H, exchangeable in D_2_O); 6.46–6.37 (m, 4H); 6.13–6.09 (m, 3H); 3.64 (br, 2H); 2.56–2.40 (m, 3H); 2.36–2.30 (m, 2H); 1.85–1.81 (m, 3H); 1.51–1.42 (m, 9H); 0.95–0.88 (m, 3H); ^13^C (DMSO_d6_): δ = 170.5; 155.1; 144.6; 140.6; 121.3; 121.2; 121.1; 120.8; 120.5; 118.0;117.9; 11.8; 110.6; 60.2; 47.2; 40.7; 40.2; 39.7; 39.4; 30.8; 29.0; 25.4; 22.2; 10.4. Mass: *m*/*z* calc C_24_H_31_N_3_O_2_ 393.2 [M + H]^+^; found 394.4. Anal (C_24_H_31_N_3_O_2_) C, H, N (Table S1).

*3*-((2*R*,6*R*,11*R*)-8-hydroxy-6,11-dimethyl-1,4,5,6-tetrahydro-2,6-methanobenzo[d]azocin-3(2H)-yl)-N-(pyridin-2-yl)propanamide (**7**). Yield = 50%; Mp = 205–206 °C; ${\left[\alpha \right]}_{D}^{25}$ = −60.4° (c 1.004, EtOH); TLC CH_2_Cl_2_/EtOH (95:5 *v*/*v*) R_f_ = 0.15; ^1^H NMR (DMSO-d_6_): δ = 10.36 (s, 1H, exchangeable in D_2_O); 8.72 (d, 1H); 8.25 (d, 1H); 7.99 (d, 1H); 7.34–7.32 (m, 1H); 6.94 (d, 1H); 6.64 (d, 1H); 6.58 (d, 1H); 3.64–3.41 (m, 6H); 3.05–2.76 (m, 4H); 1.98–183 (m, 2H); 1.28 (s, 3H); 0.78 (d, 3H). ^13^C (DMSO-d_6_): δ = 177.7; 157.0; 152.0; 145.1; 141.5; 136.2; 128.9; 127.0; 124.6; 114.3; 112.7; 110.4; 55.0; 49.6; 46.1; 43.4; 41.9; 40.3; 39.9; 24.2; 22.3; 13.5. Mass: *m*/*z* calc C_22_H_27_N_3_O_2_ 365.2 [M + H]^+^; found 366.2. Anal (C_22_H_27_N_3_O_2_) C, H, N (Table S1).

*3*-((2*S*,6*S*,11*S*)-8-hydroxy-6,11-dimethyl-1,4,5,6-tetrahydro-2,6-methanobenzo[d]azocin-3(2H)-yl)-N-(pyridin-2-yl)propanamide (**8**). Yield = 40%; Mp = 205–208 °C; ${\left[\alpha \right]}_{D}^{25}$ = +59.6° (c 1.003, EtOH); TLC CH_2_Cl_2_/EtOH (95:5 *v*/*v*) R_f_ = 0.17; ^1^H NMR (DMSO-d_6_): δ = 10.40 (s, 1H, exchangeable in D_2_O); 8.75 (d, 1H); 8.29 (d, 1H); 8.03 (d, 1H); 7.38–7.36 (m, 1H); 6.98 (d, 1H); 6.66 (d, 1H); 6.63 (d, 1H); 3.41–3.25 (m, 6H); 3.09–2.80 (m, 4H); 2.02–1.87 (m, 2H), 1.32 (s, 3H); 0.82 (d, 3H). ^13^C (DMSO-d_6_): δ = 177.4; 156.2; 152.0; 144.7; 141.1; 135.8; 128.5; 126.6; 124.3; 113.9; 112.3; 108.5; 54.6; 50.0; 46.1; 43.0; 39.9; 39.4; 39.1; 23.8; 21.9; 13.1. Mass: *m*/*z* calc C_22_H_27_N_3_O_2_ 365.2 [M + H]^+^; found 366.2. Anal (C_22_H_27_N_3_O_2_) C, H, N (Table S1).

### 4.4. MOR, DOR, and KOR Radioligand Binding Assays

The radioligand binding assays and the data analysis were performed as previously reported [23].

### 4.5. Radioligand Binding Assays for σ1R and σ2R 

The radioligand binding assays and the data analysis were performed as previously reported [24].

### 4.6. Isolated Organs: Mouse Vas Deferens (MVD)

Animals. Male NMRI mice (Toxicoop Zrt., Budapest, Hungary) weighing 35–45 g were kept in groups in a temperature- and humidity-controlled room under a 12 h light/dark cycle and with food and water available ad libitum. Each animal was used for one experiment. Experimental procedures were approved by the local ethical committee (IACUC) and conducted in accordance with international guidelines as well as the European Communities Council Directive and National Regulations (CEE Council 86/609 and DL 116/92). 

Isolated mouse vas deferens. The preparation of MVD was carried out as previously described by Hughes et al. [25] with minor modifications. Briefly, vasa deferentia were removed from the mice and unsheathed from the surrounding fat, connective tissue, and blood vessels. They were then suspended between an upper (ring) and a lower (straight) electrode positioned at 5.5 cm, in 5 mL organ baths containing Krebs solution (concentrations in mM: NaCl, 118.0; NaHCO_3_, 25.0; KCl, 4.7; KH_2_PO4, 1.2; glucose, 11.0; CaCl_2_, 2.5; and MgSO_4_, 1.2) aerated with a gas mixture of 95% O_2_ and 5% CO_2_ and at a constant temperature of 36 °C. The upper end of the isolated organ was attached to a transducer using a thread and connected to a computer via an amplifier (PowerLab 4/20, ADInstruments, Castle Hill, NSW, Australia). The resting tension was adjusted to 0.1 g. Electrical field stimulation was applied through trains of 10 Hz with 10 rectangular impulses at a 1 ms pulse width, 9 V/cm (i.e., supramaximal intensity), repeated with 0.1 Hz (Stimulator 88, Grass Medical Instruments, Quincy, MA, USA). 

Experimental paradigms. The experimental design of MVD required a 50 min equilibration period under electrical field stimulation, during which Krebs solution was changed every 5 min. After the equilibration period, test compounds were added cumulatively, allowing a minimum of 2 min between doses. To determine the test compounds’ dissociation constant, K_e_, vasa deferentia were preincubated with the antagonist naloxone, and without washing a single concentration of the agonist test, the compound was added.

Data analysis. The efficacy of each test compound to inhibit electrically evoked contraction was measured as the percentage change from baseline. The 50% inhibiting concentrations, IC_50_, were determined by nonlinear regression (Hill slope, three parameters) of logarithmic dose–response curves using GraphPad Prism 8.01 software (San Diego, CA, USA). Each statistical analysis had a minimum of *n* = 8 independent experiments. Data are presented as the mean ± SEM. K_e_ values were calculated using dose ratio and the single-dose method [26]. Statistical analysis was performed with one-way ANOVA, followed by the Newman–Keuls post hoc test. Results were considered significantly different when the *p*-value was less than 0.05. 

### 4.7. In Vivo Pharmacology 

Thermal acute pain model (tail-flick test). Male Swiss CD1 mice (Envigo Laboratories, S. Pietro al Natisone (UD)) weighing 25–30 g were housed at six per cage, kept at a constant room temperature (25 ± 1 °C), under a 12:12 h light-and-dark cycle with food and water ad libitum. Each mouse was used for only one experiment. Experimental procedures were approved by the local ethical committee (IACUC) and conducted in accordance with international guidelines as well as the European Communities Council Directive and National Regulations (CEE Council 86/609 and DL 116/92). The radiant heat tail-flick test consists of irradiation of the lower third of the tail with an infrared source (Ugo Basile, Comerio, Italy) [27]. The day before the experiment, performed at room temperature (25 ± 1 °C), mice were habituated to the procedure for measuring the nociception threshold. The basal predrug latency was established between 3 and 6 s and was calculated as the average of the first three measurements, which were performed at 5 min intervals. A cutoff latency of 15 s was established to minimize damage to the tail. All tested compounds were dissolved in pyrogen-free isotonic saline (Baxter Healthcare, Deerfield, IL, USA) and DMSO (5%) and were administered to mice i.p. Post-treatment tail-flick latencies (TFLs) were determined at 15, 30, 45, 60, and 90 min after i.p. injection. 

Inflammatory pain model (CFA-evoked hyperalgesia). Male Wistar rats (190–250 g) (Toxicoop Zrt., Budapest, Hungary) under brief isoflurane (Willy Rüsch GmbH, Böblingen, Germany) anesthesia received i.pl. injection of 0.15 mL complete Freund’s adjuvant (CFA) (source), a water-in-oil emulsion of killed mycobacterium, into the right hind paw. The experimental procedures were performed as described previously [28].

Experimental paradigms of CFA-evoked hyperalgesia. The measurement of the effect of the test compound was carried out 4 days following i.pl. CFA injection as described previously [28]. Briefly, the baseline (pretest compound) of the paw pressure threshold (PPT) of both inflamed and noninflamed paws was determined. Next, the drug or vehicle was s.c. administered at a volume of 5 mL/kg body weight. Then, the response threshold to mechanical pressure stimulation of inflamed and noninflamed paws was given as paw pressure thresholds (PPTs) and measured at 30 and 60 min post administration using an arbitrary cutoff weight of twice the baseline.

## 5. Conclusions

Nowadays, the management of pain is challenging, and available therapies with opioid-based analgesics are being used to manage acute and chronic mild–severe pain, although they are often associated with serious side effects, such as respiratory depression, constipation, and addiction liability. Moreover, with respect to current clinical applications, opioids remain the mainstay for the treatment of cancer pain [29,30]. Therefore, to optimize their tolerability profiles, several researchers in medicinal chemistry continue to study novel ligands and innovative approaches.

Here, a series of LP1 analogs were synthesized for SAR studies concerning the *N*-substituent of the benzomorphan skeleton, evaluating how the electronic characteristics of the aromatic ring and their relative distance from the basic nitrogen affect the opioid receptors’ interaction. Opioid receptor binding assays showed that electron-rich and electron-deficient rings were favorable for MOR affinity and selectivity over DOR when the propanamide group was the linker, with a lower KOR selectivity with respect to LP1. However, the different electronic properties of the aromatic ring in the *N*-substituent of compounds **3** and **7** reflected in a different signaling pathway at MOR being the former an antagonist and the latter an agonist as potent as the lead compound and DAMGO. The MOR agonist profile was also confirmed in vivo in both nociceptive and inflammatory animal pain models.

As continuous searching in novel and improved analgesics is a goal in medicinal chemistry research, these findings allowed us to deepen our SARs, improving the understanding of the chemical features of the *N*-substituent favorable for MOR and KOR interaction and discriminating DOR. Indeed, the structural modifications made at the *N*-substituent change the pharmacodynamic profile from multitarget MOR agonist/DOR antagonist (LP1) to MOR/KOR agonist or MOR antagonist (compounds **7** and **3**). In our research group, further investigation pointed at the *N*-substituent properties of (−)-*cis*-*N*-normetazocine is ongoing.

## Figures and Tables

**Table 1:** Opioid and σ receptor binding assays for compounds **3**–**8**.

Compound	*N*-Substituent	K_i_ (nM) ± SD ^a^					K_i_ Ratios	
		MOR	DOR	KOR	σ1R	σ2R	$\frac{DOR}{MOR}$	$\frac{KOR}{MOR}$
**3**	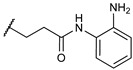	5.96 ± 0.08	155 ± 1.5	93 ± 0.63	>10,000	>10,000	26.0	15.6
**4**	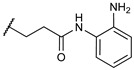	408 ± 11	3120 ± 73	154 ± 6	729 ± 168	>10,000	7.6	0.38
**5**	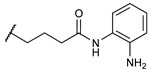	479 ± 10	1028 ± 16	970 ± 21	>10,000	>10,000	2.15	2.0
**6**	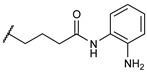	810 ± 15	4190 ± 60	2455 ± 11	857 ± 212	>10,000	5.2	1.1
**7**	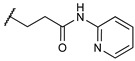	1.49 ± 0.24	138 ± 5	64 ± 7	>10,000	>10,000	92.6	42.9
**8**	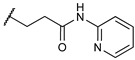	563 ± 23	>10,000	1379 ± 20	2511 ± 737	>10,000	-	2.4
LP1 ^b^		0.83 ± 0.05	29.1 ± 1	110 ± 6	149 ± 4.00	1680	35.1	132.5
DAMGO		1.16 ± 0.10	—	—	—	—		
Naltrindole		—	1.13 ± 0.10	—	—	—		
(−)-U50,488		—	—	1.10 ± 0.21	—	—		
Haloperidol		—	—	—	2.6 ± 0.4	77 ± 18		
(+)-Pentazocine		—	—	—	4.3 ± 0.5	1465 ± 224		
DTG		—	—	—	124 ± 19	18 ± 1		
BD-1063		—	—	—	14 ± 2.7	204 ± 31		

^a^ Each value is the mean ± SD of at least two experiments performed in duplicate. Reference compounds were tested with the same membrane homogenates. ^b^ Reference [3].

**Table 2:** The 50% inhibitory concentrations (IC_50_) and affinity values (K_e_) of compound **7** and compound **3** compared with DAMGO (selective MOR agonist), DPDPE (selective DOR agonist), and EKC (selective KOR agonist) on MOR, DOR, and KOR in isolated MVD biological assay. ^a^ immeasurable. *n* = 5–9.

Compound	IC_50_ ± SEM (nM)	K_e_ ± SEM (nM)		
		Naloxone (MOR)	Naltrindole (DOR)	Nor-BNI (KOR)
Compound **7**	66.88 ± 0.38	2.42 ± 0.45	^a^	68.18 ± 15.98
DAMGO	66.86 ± 1.52	2.75 ± 0.40	8.24 ± 3.12	6.24 ± 2.09
DPDPE	9.97 ± 0.44	4.28 ± 0.52	0.13 ± 0.03	2.57 ± 1.81
EKC	14.05 ± 0.69	8.82 ± 0.52	11.47 ± 5.35	1.76 ± 0.35

## Data Availability

The data presented in this study are available on request from the corresponding author.

## Supplementary Materials

The following supporting information can be downloaded at https://www.mdpi.com/article/10.3390/molecules28124827/s1, Figure S1: ^1^H-NMR (200 MHz, CDCl_3_) spectrum of compound **3**; Figure S2: ^13^C-NMR (50 MHz, CDCl_3_) spectrum of compound **3**; Figure S3: MALDI-TOF spectrum of compound **3**; Figure S4: ^1^H-NMR (200 MHz, CDCl_3_) spectrum of compound **4**; Figure S5: ^13^C-NMR (50 MHz, CDCl_3_) spectrum of compound **4**; Figure S6: MALDI-TOF spectrum of compound **4**; Figure S7: ^1^H-NMR (500 MHz, DMSO-*d*_6_) spectrum of compound **5**; Figure S8: ^13^C-NMR (50 MHz, DMSO-*d*_6_) spectrum of compound **5**; Figure S9: MALDI-TOF spectrum of compound **5**; Figure S10: ^1^H-NMR (500 MHz, DMSO-*d*_6_) spectrum of compound **6**; Figure S11: ^13^C-NMR (50 MHz, DMSO-*d*_6_) spectrum of compound **6**; Figure S12: MALDI-TOF spectrum of compound **6**; Figure S13: ^1^H-NMR (200 MHz, DMSO-*d*_6_) spectrum of compound **7**; Figure S14: ^13^C-NMR (125 MHz, DMSO-*d*_6_) spectrum of compound **7**; Figure S15: MALDI-TOF spectrum of compound **7**; Figure S16: ^1^H-NMR (200 MHz, DMSO-*d*_6_) spectrum of compound **8**; Figure S17: ^13^C-NMR (125 MHz, DMSO-*d*_6_) spectrum of compound **8**; Figure S18: MALDI-TOF spectrum of compound **8**; Table S1: Elemental analysis data for compounds **3**–**8**.

## Sample Availability

Samples of all synthesized compounds are available from the authors.
