# Supplementary material for: New Insights into the Opioid Analgesic Profile of cis-(−)-N-Normetazocine-derived Ligands
Source: Molecules. 2023 Jun 17;28(12):4827. doi: 10.3390/molecules28124827 (PMC10301092; doi:10.3390/molecules28124827)
Supplement: Supplementary file 1 [file molecules-28-04827-s001.zip › molecules-2425867-supplementary.pdf]

Supporting Information

New Insights into the Opioid Analgesic Profile of *cis*-(−)-*N*-Normetazocine-derived Ligands

Giuliana Costanzo [1], Rita Turnaturi [2], Carmela Parenti [3], Salvatore Spoto [3], Silvia Piana [2], Maria Dichiara [2], Chiara Zagni [2], Anna Rita Galambos [4], Nariman Essmat [4], Agostino Marrazzo [2], Emanuele Amata [2], Mahmoud Al-Khrasani [4,*] and Lorella Pasquinucci [2,*]

[1] Department of Biomedical and Biotechnological Sciences, University of Catania, Via Santa Sofia 97, 95123 Catania, Italy; giuliana.costanzo93@gmail.com

[2] Department of Drug and Health Sciences, Medicinal Chemistry Section, University of Catania, Viale A. Doria 6, 95125 Catania, Italy; rita.turnaturi@unict.it (R.T.); silviapiana@outlook.com (S.P.); mariadic.md@gmail.com (M.D.); chiara.zagni@unict.it (C.Z.); marrazzo@unict.it (A.M.); eamata@unict.it (E.A.)

[3] Department of Drug and Health Sciences, Section of Pharmacology and Toxicology, University of Catania, Viale A. Doria 6, 95125 Catania, Italy; cparenti@unict.it (C.P.); salvospoto12@icloud.com (S.S.)

[4] Department of Pharmacology and Pharmacotherapy, Faculty of Medicine, Semmelweis University, Nagyvárad tér 4, H-1089 Budapest, Hungary; galambos.anna@phd.semmelweis.hu (A.R.G.); nariman.gomaa@phd.semmelweis.hu (N.E.)

* Correspondence: al-khrasani.mahmoud@med.semmelweis-univ.hu (M.A.-K.); lpasquin@unict.it (L.P.)

Table of contents

NMR and MS spectra of compound **3**. (Figures S1-S3)	S3-S4
NMR and MS spectra of compound **4**. (Figures S4-S6)	S5-S6
NMR and MS spectra of compound **5**. (Figures S7-S9)	S7-S8
NMR and MS spectra of compound **6**. (Figures S10-S12)	S9-S10
NMR and MS spectra of compound **7**. (Figures S13-S15)	S11-S12
NMR and MS spectra of compound **8**. (Figures S16-S18)	S13-S14
Table S1. Elemental analysis data for compounds **3-8**.	S15

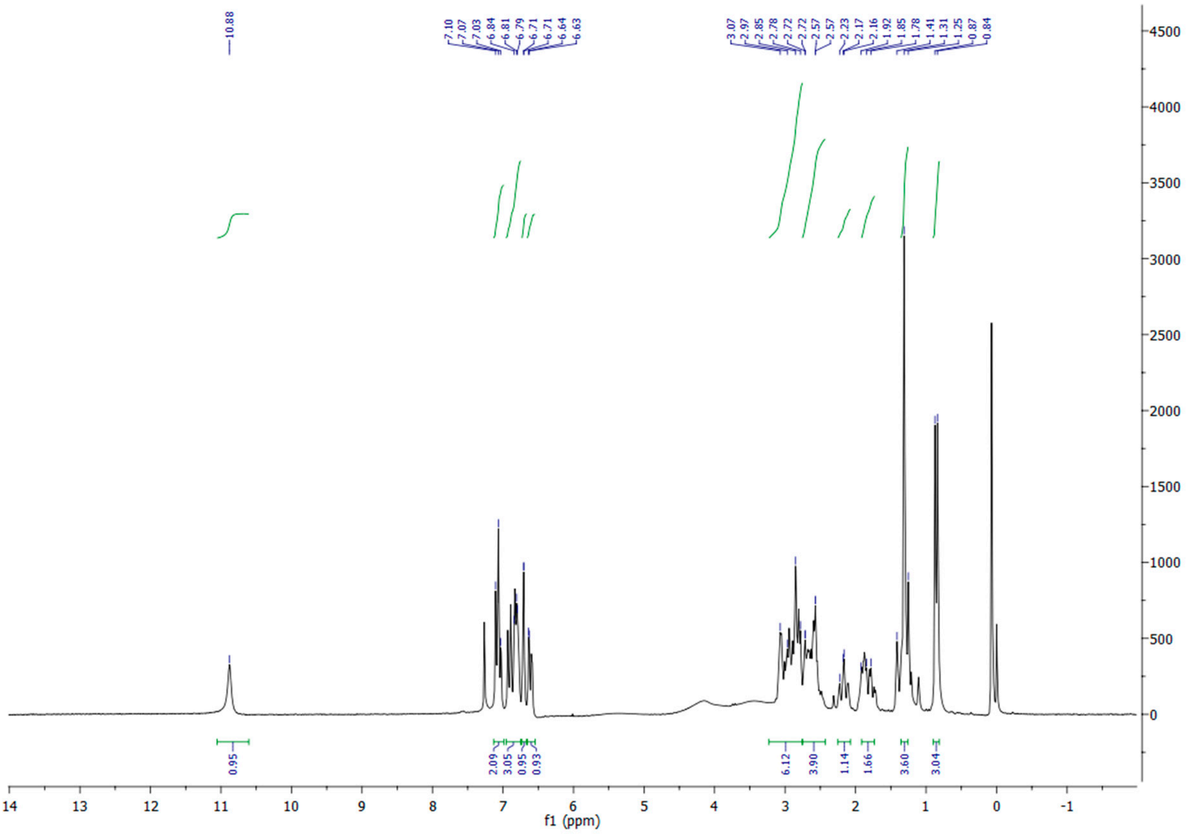

Figure S1. ^{1}H-NMR (200 MHz, $CDCl_3$) spectrum of compound **3**.

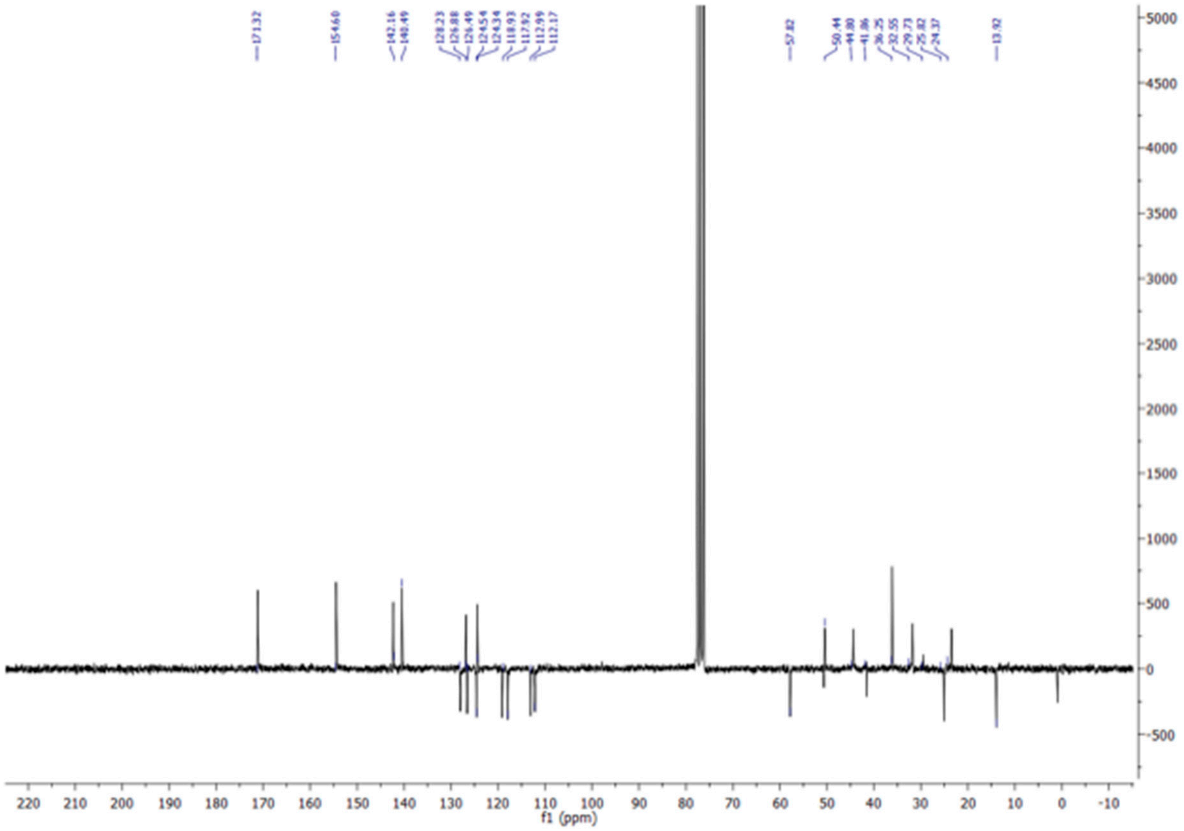

Figure S2. ^{13}C-NMR (200 MHz, $CDCl_3$) spectrum of compound **3**.

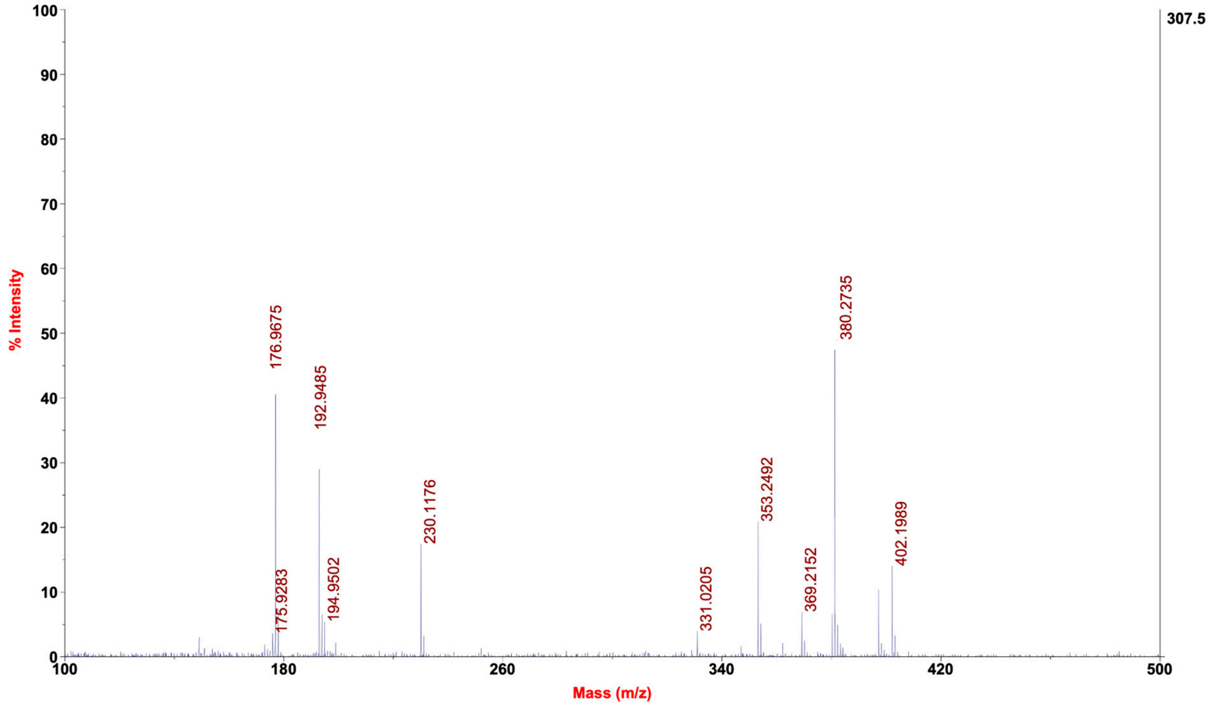

Figure S3. MALDI spectrum of compound **3**.

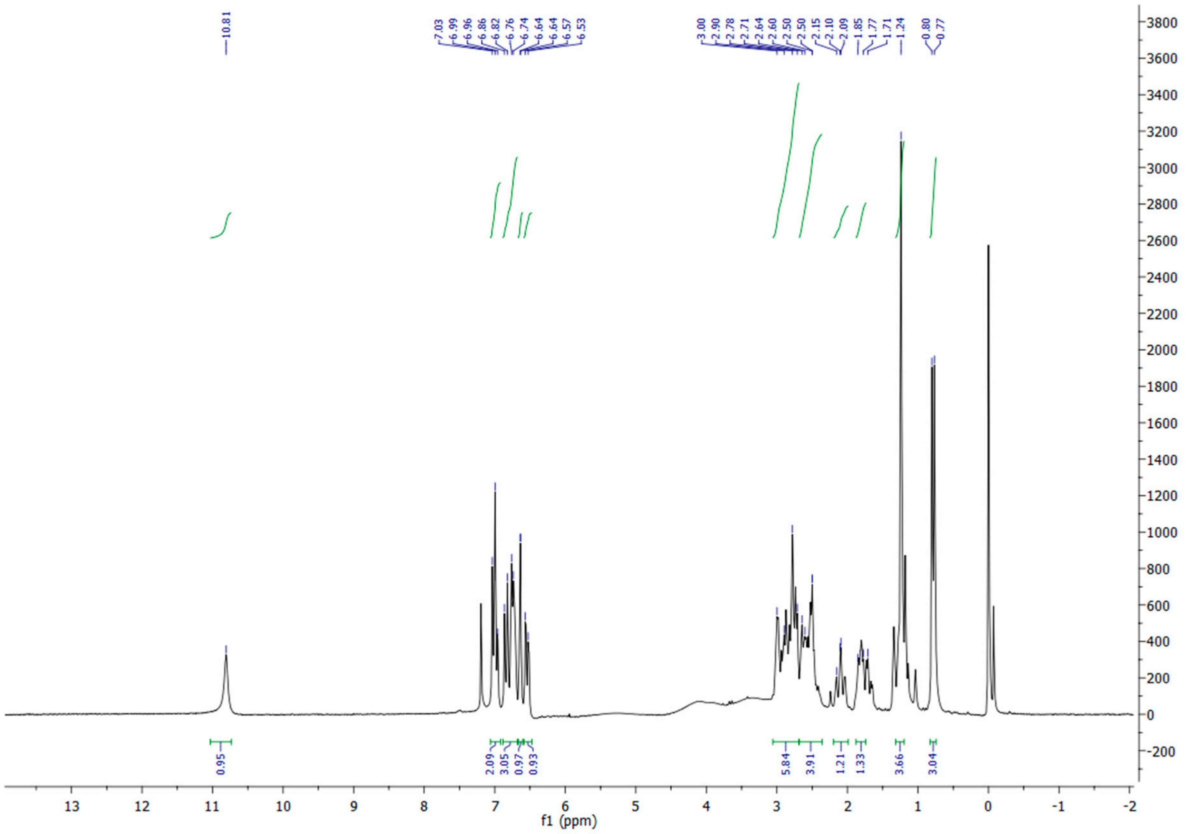

Figure S4. ^{1}H-NMR (200 MHz, $CDCl_3$) spectrum of compound **4**.

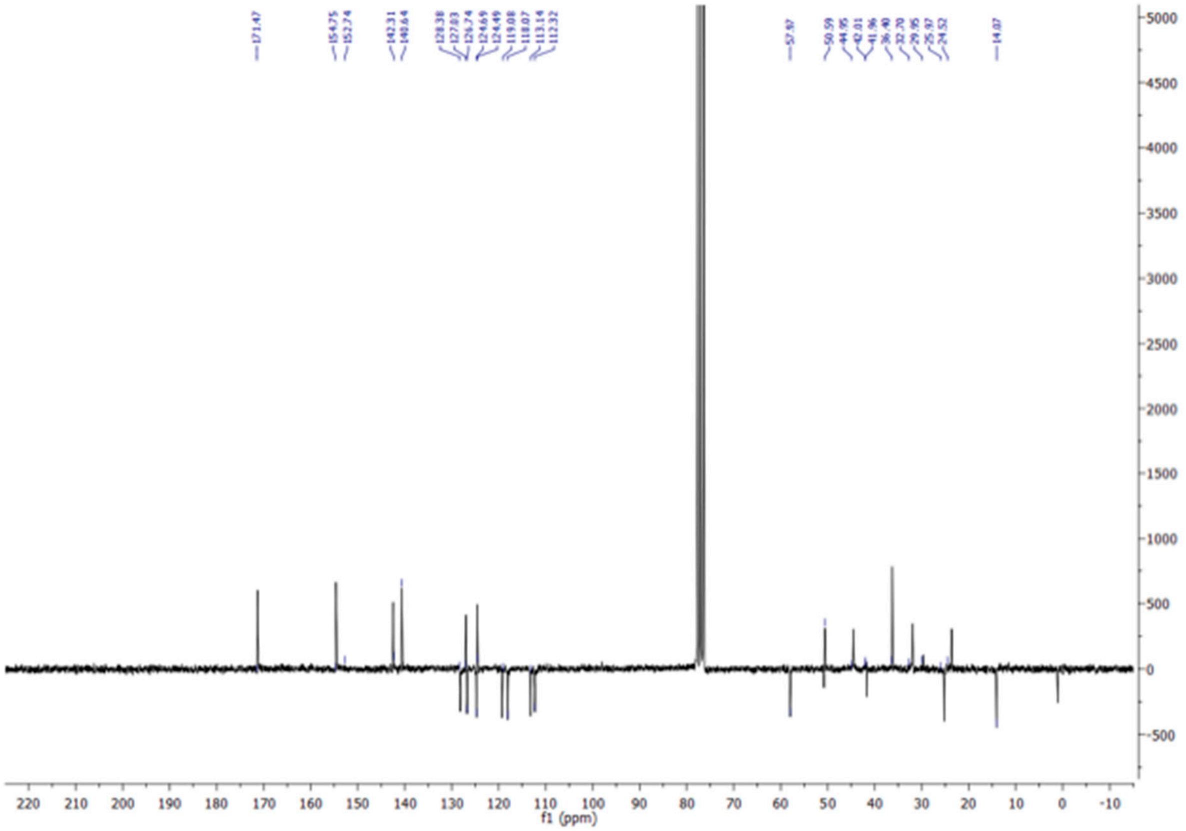

Figure S5. ^{13}C-NMR (200 MHz, $CDCl_3$) spectrum of compound **4**.

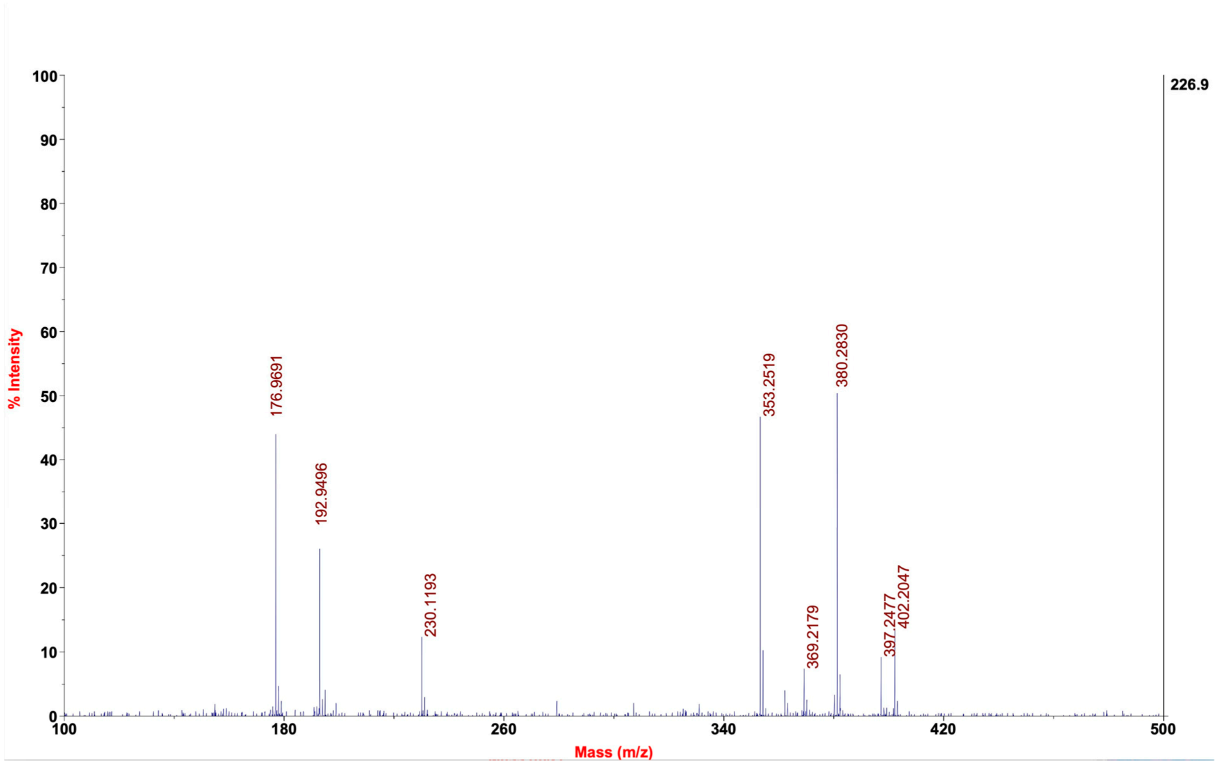

Figure S6. MALDI spectrum of compound **4**.

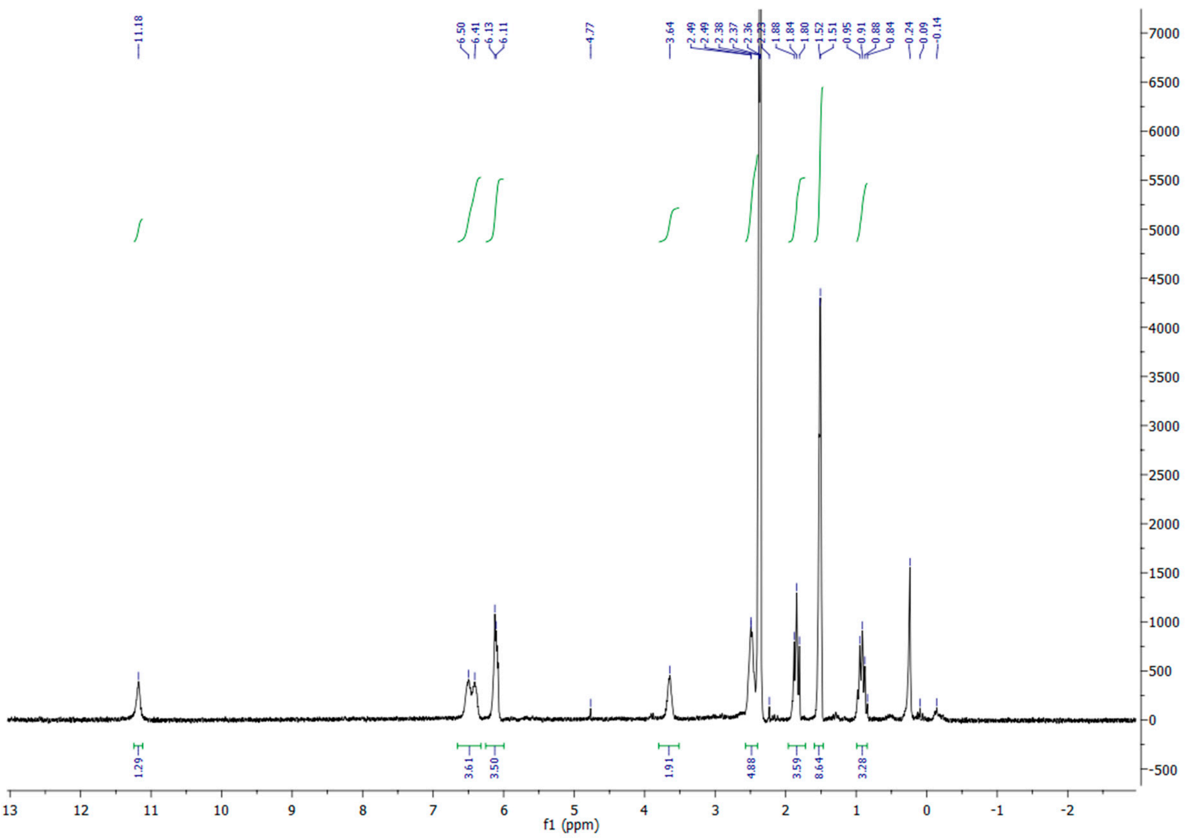

Figure S7. ^{1}H-NMR (200 MHz, DMSO-d_6) spectrum of compound **5**.

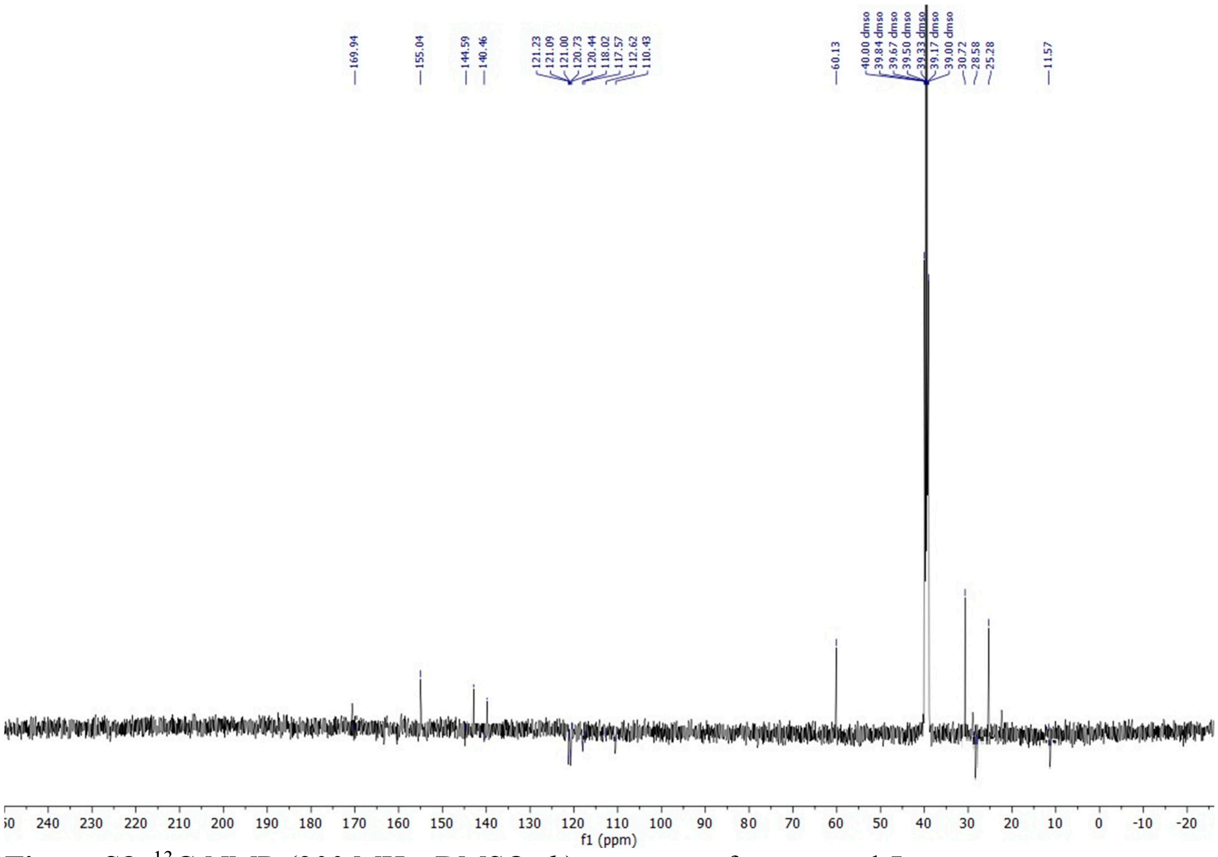

Figure S8. ^{13}C-NMR (200 MHz, DMSO-d_6) spectrum of compound **5**.

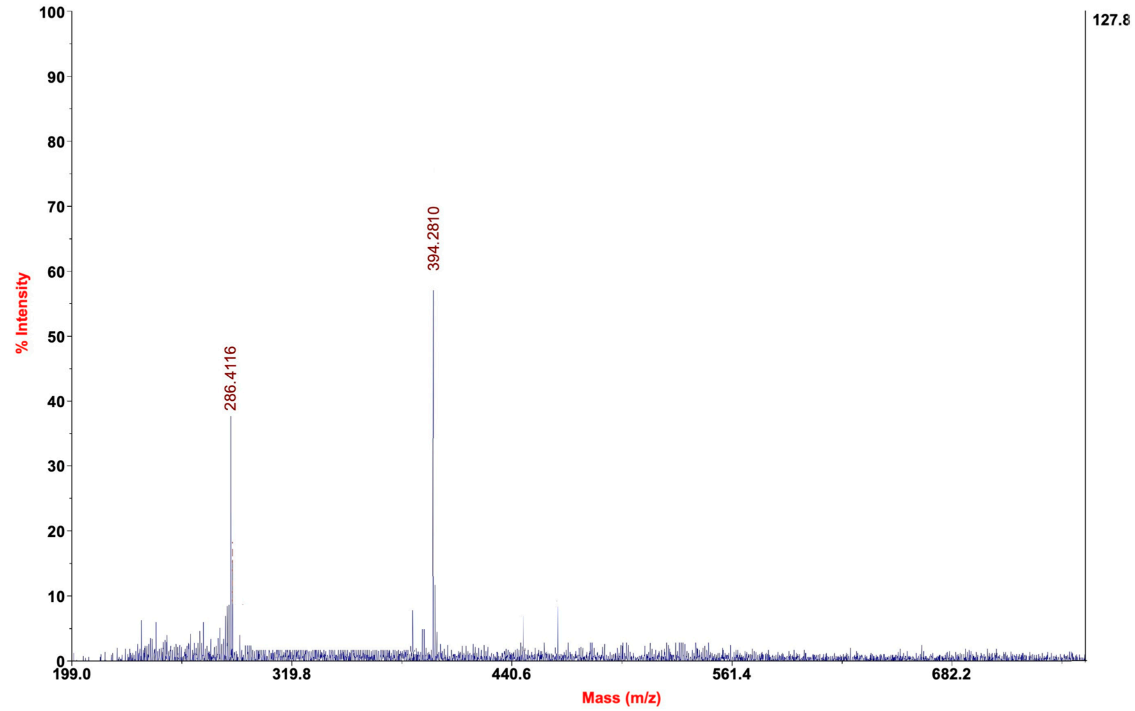

Figure S9. MALDI spectrum of compound **5**.

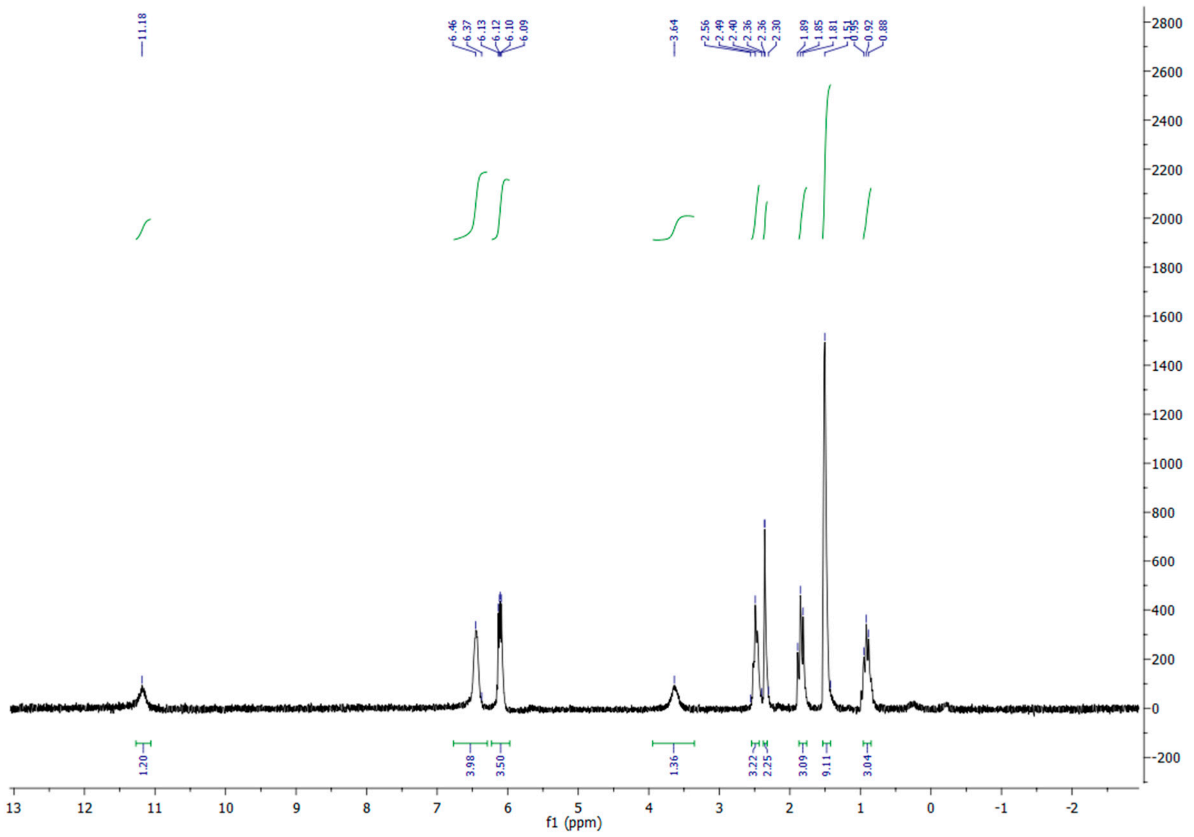

Figure S10. ^{1}H-NMR (200 MHz, DMSO-d_6) spectrum of compound **6**.

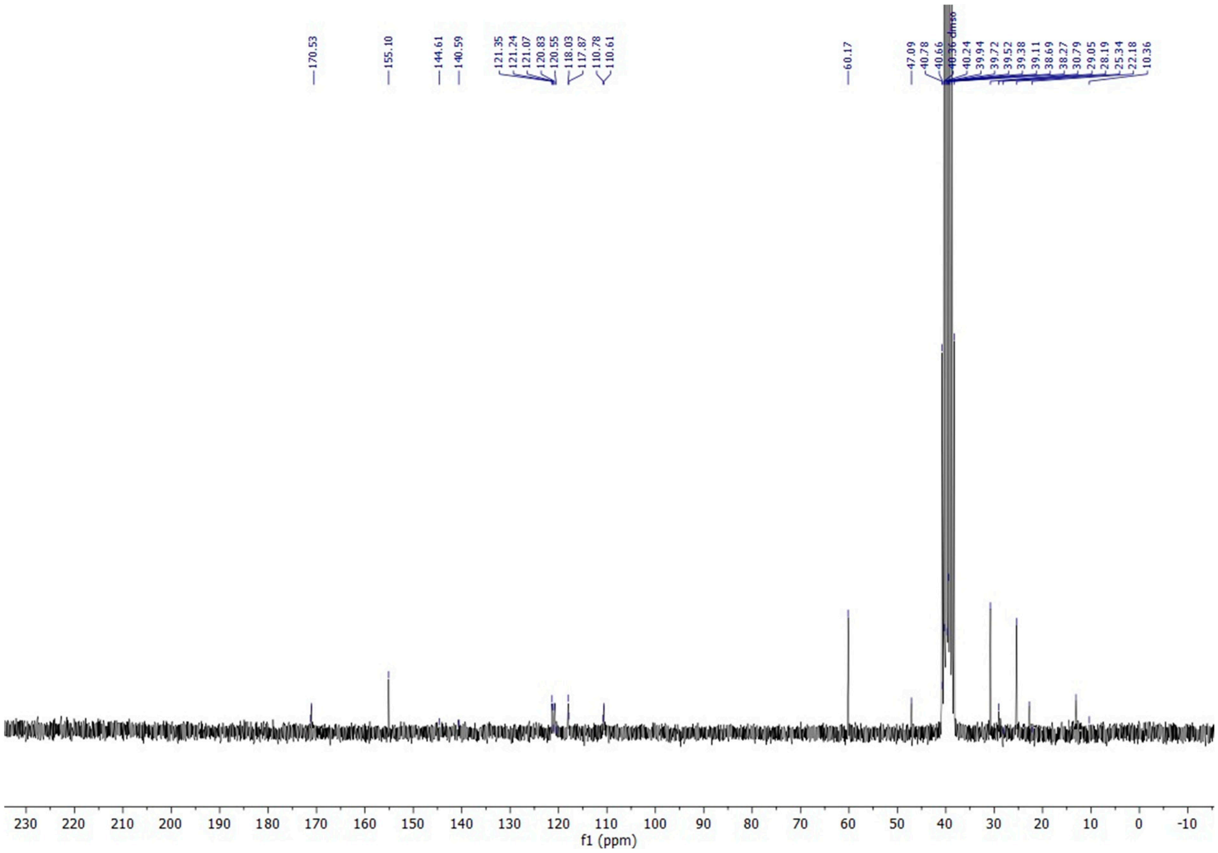

Figure S11. ^{13}C-NMR (200 MHz, DMSO-d_6) spectrum of compound **6**.

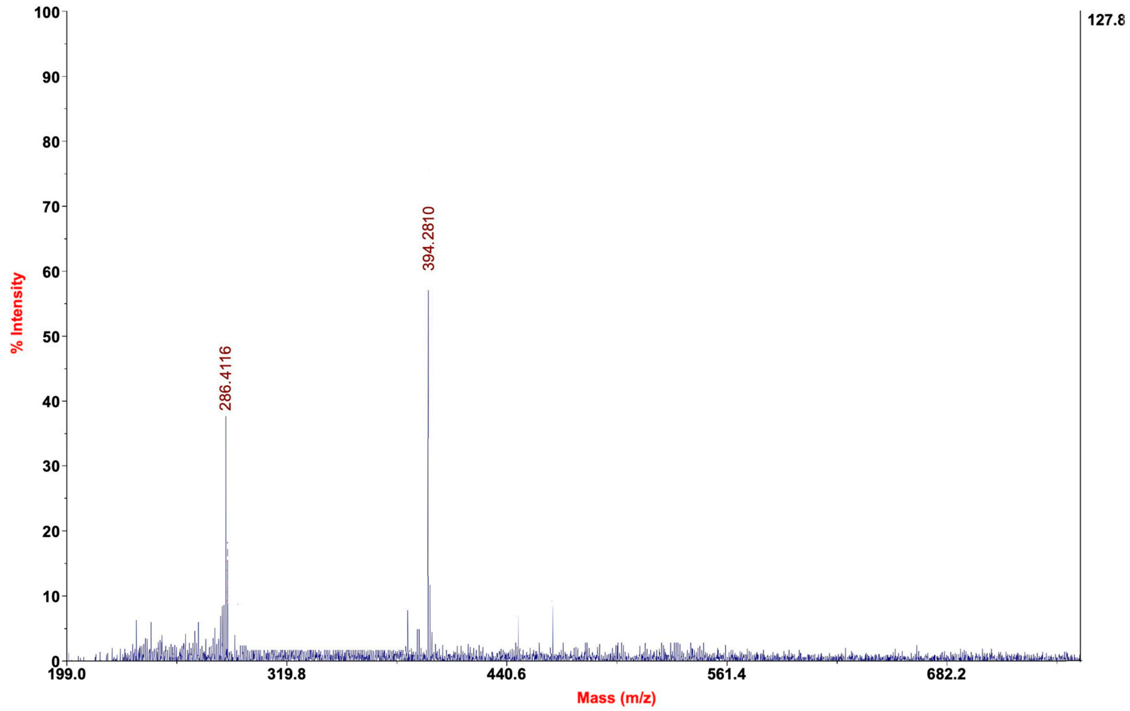

Figure S12. MALDI spectrum of compound **6**.

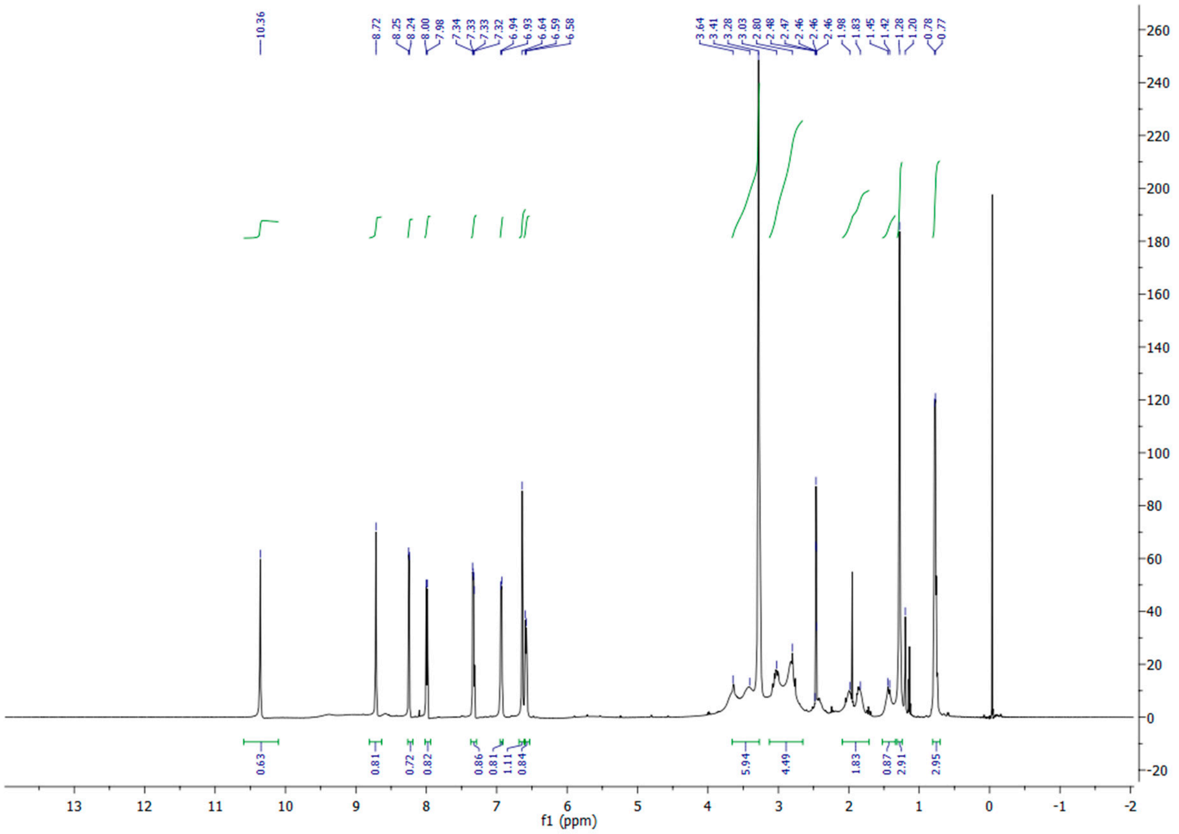

Figure S13. ^{1}H-NMR (200 MHz, DMSO-d_6) spectrum of compound **7**.

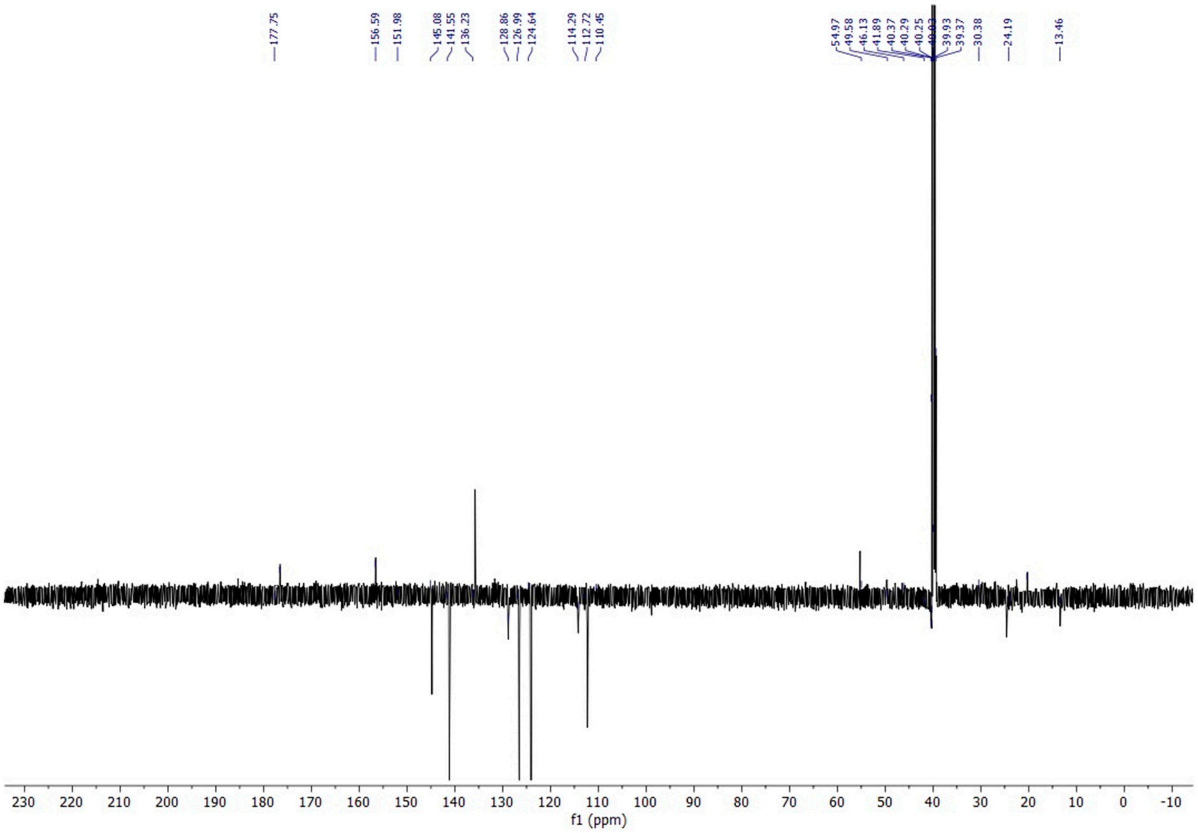

Figure S14. ^{13}C-NMR (200 MHz, DMSO-d_6) spectrum of compound **7**.

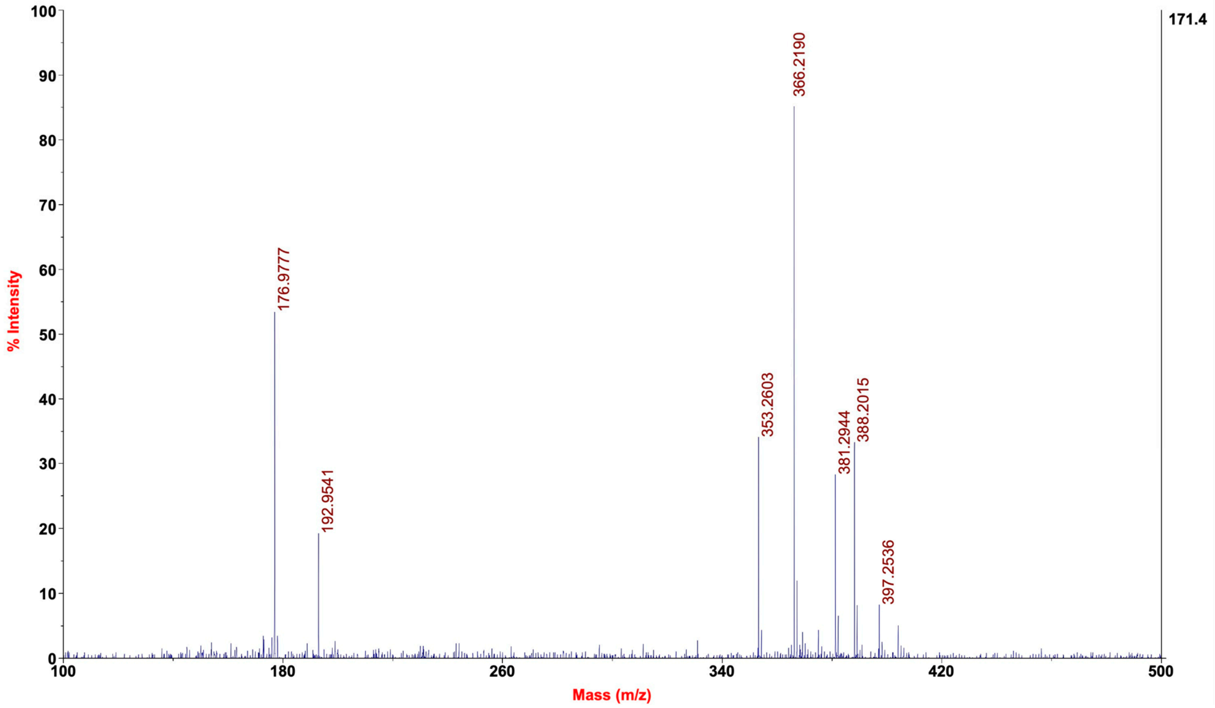

Figure S15. MALDI spectrum of compound **7**.

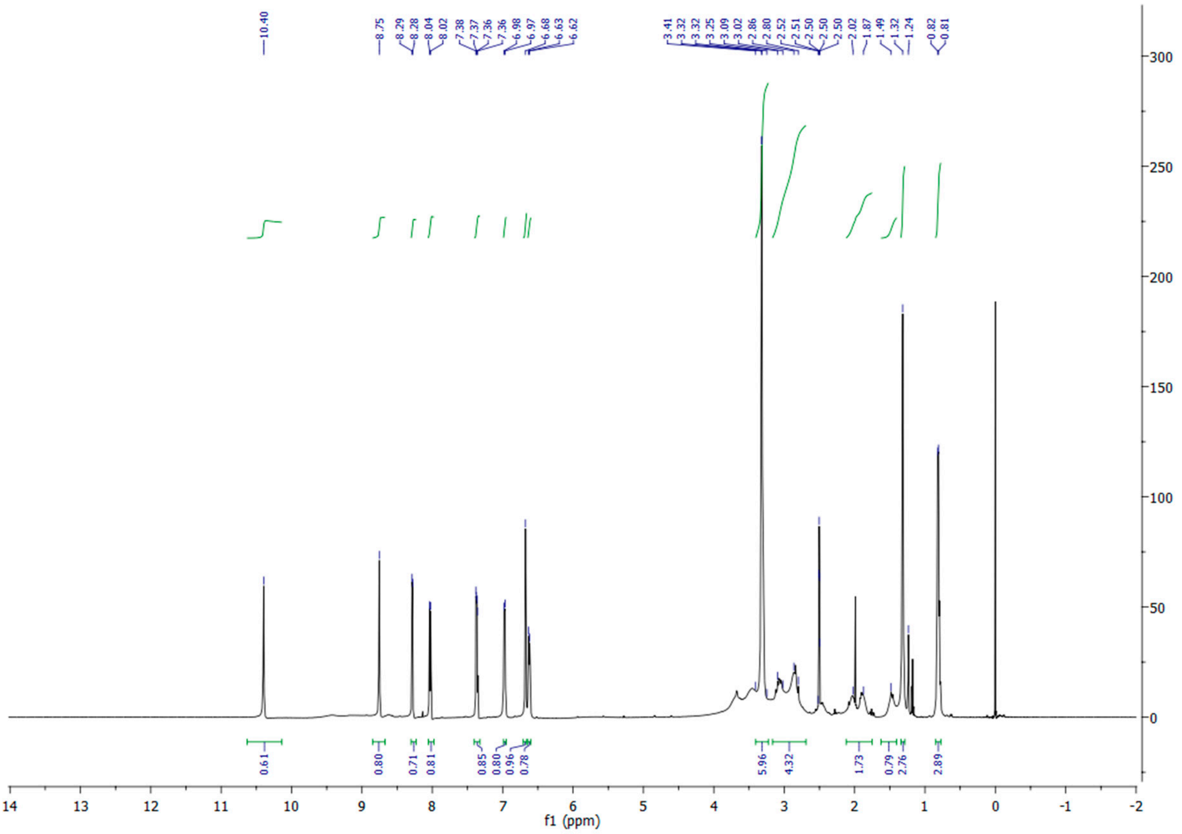

Figure S16. ^{1}H-NMR (200 MHz, DMSO-d_6) spectrum of compound **8**.

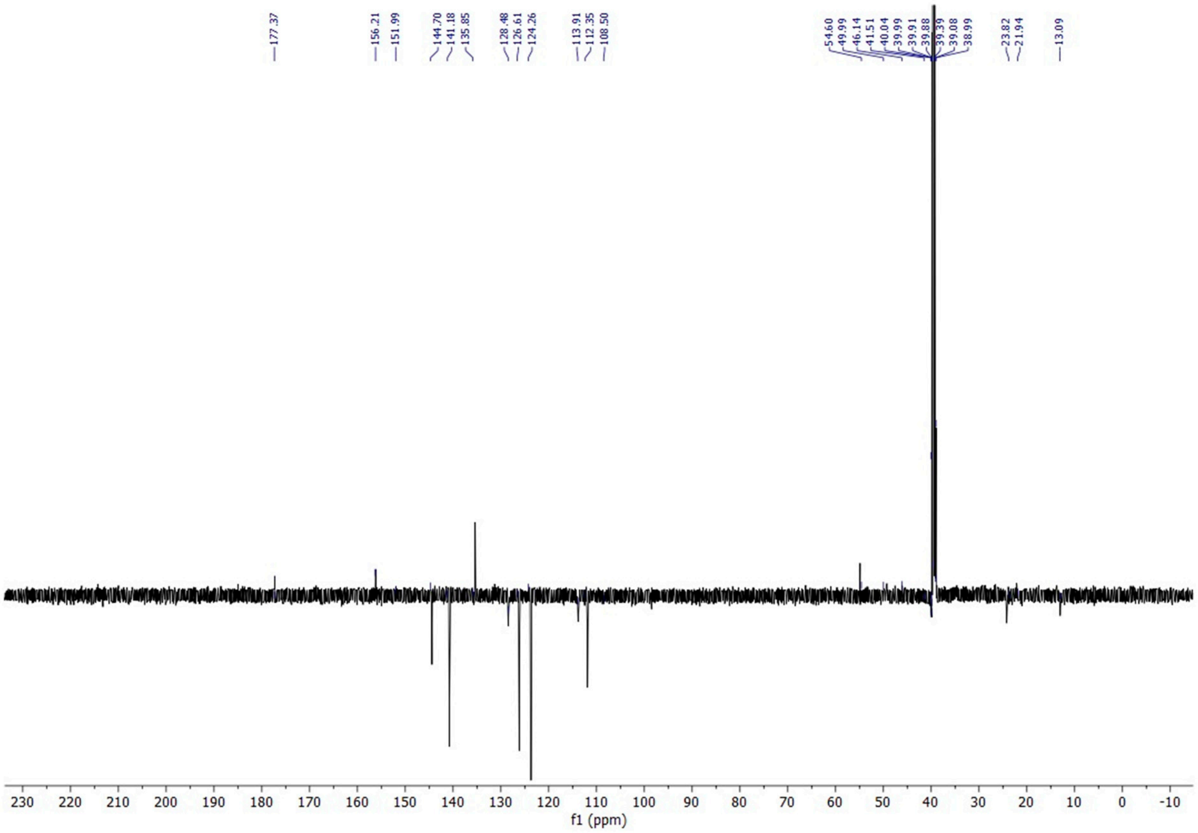

Figure S17. ^{13}C-NMR (200 MHz, DMSO-d_6) spectrum of compound **8**.

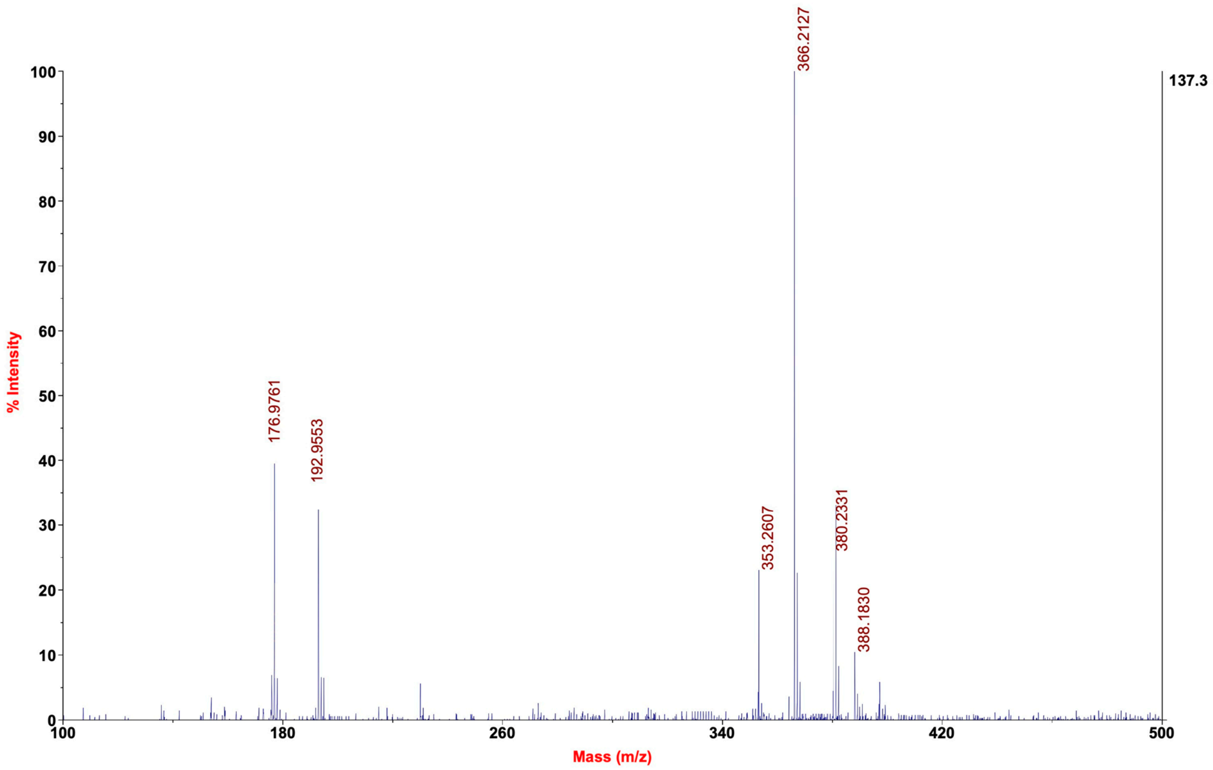

Figure S18. MALDI spectrum of compound **8**.

Table S1. Elemental analysis data for compounds **3–8**.

			Calcd			Found		
**Compd**	**Formula**	**MW**	**C**	**H**	**N**	**C**	**H**	**N**
**3**	$C_{23}H_{29}N_3O_2$	379.49	72.79	7.70	11.07	72.82	7.73	11.04
**4**	$C_{23}H_{29}N_3O_2$	379.49	72.79	7.70	11.07	72.78	7.74	11.02
**5**	$C_{24}H_{31}N_3O_2$	393.52	73.25	7.94	10.68	73.27	7.96	10.63
**6**	$C_{24}H_{31}N_3O_2$	393.52	73.25	7.94	10.68	73.23	7.97	10.65
**7**	$C_{22}H_{27}N_3O_2$	365.46	72.30	7.45	11.50	72.32	7.47	11.48
**8**	$C_{22}H_{27}N_3O_2$	365.46	72.30	7.45	11.50	72.34	7.45	11.46
